# A Single Multilocus Sequence Typing (MLST) Scheme for Seven Pathogenic *Leptospira* Species

**DOI:** 10.1371/journal.pntd.0001954

**Published:** 2013-01-24

**Authors:** Siriphan Boonsilp, Janjira Thaipadungpanit, Premjit Amornchai, Vanaporn Wuthiekanun, Mark S. Bailey, Matthew T. G. Holden, Cuicai Zhang, Xiugao Jiang, Nobuo Koizumi, Kyle Taylor, Renee Galloway, Alex R. Hoffmaster, Scott Craig, Lee D. Smythe, Rudy A. Hartskeerl, Nicholas P. Day, Narisara Chantratita, Edward J. Feil, David M. Aanensen, Brian G. Spratt, Sharon J. Peacock

**Affiliations:** 1 Mahidol-Oxford Tropical Medicine Research Unit, Faculty of Tropical Medicine, Mahidol University, Bangkok, Thailand; 2 Department of Microbiology and Immunology, Faculty of Tropical Medicine, Mahidol University, Bangkok, Thailand; 3 Department of Infection and Tropical Medicine, Birmingham Heartlands Hospital, West Midlands, United Kingdom; 4 Pathogen Genomics, Wellcome Trust Sanger Institute, Cambridge, United Kingdom; 5 Department of Leptospirosis, National Institute for Communicable Disease Control and Prevention, Chinese Center for Disease Control and Prevention, Beijing, People's Republic of China; 6 Department of Bacteriology, National Institute of Infectious Diseases, Tokyo, Japan; 7 Laboratory of Wildlife Biology and Medicine, Graduate School of Veterinary Medicine, Hokkaido University, Sapporo, Japan; 8 National Center for Emerging and Zoonotic Infectious Diseases, Centers for Diseases Control and Prevention, Atlanta, Georgia, United States of America; 9 WHO/FAO/OIE Collaborating Center for Reference and Research on Leptospirosis, Queensland Health Forensic and Scientific Services, Health Services Support Agency, Queensland Health, Brisbane, Queensland, Australia; 10 WHO/FAO/OIE and National Leptospirosis Reference Centre, KIT Biomedical Research, Royal Tropical Institute, Amsterdam, The Netherlands; 11 Center for Tropical Medicine, Nuffield Department of Clinical Medicine, University of Oxford, Churchill Hospital, Oxford, United Kingdom; 12 Department of Biology and Biochemistry, University of Bath, Bath, United Kingdom; 13 Department of Infectious Disease Epidemiology, Imperial College London, London, United Kingdom; 14 Department of Medicine, University of Cambridge, Addenbrooke's Hospital, Cambridge, United Kingdom; Institut Pasteur, France

## Abstract

**Background:**

The available *Leptospira* multilocus sequence typing (MLST) scheme supported by a MLST website is limited to *L. interrogans* and *L. kirschneri*. Our aim was to broaden the utility of this scheme to incorporate a total of seven pathogenic species.

**Methodology and Findings:**

We modified the existing scheme by replacing one of the seven MLST loci (*fadD* was changed to *caiB*), as the former gene did not appear to be present in some pathogenic species. Comparison of the original and modified schemes using data for *L. interrogans* and *L. kirschneri* demonstrated that the discriminatory power of the two schemes was not significantly different. The modified scheme was used to further characterize 325 isolates (*L. alexanderi* [n = 5], *L. borgpetersenii* [n = 34], *L. interrogans* [n = 222], *L. kirschneri* [n = 29], *L. noguchii* [n = 9], *L. santarosai* [n = 10], and *L. weilii* [n = 16]). Phylogenetic analysis using concatenated sequences of the 7 loci demonstrated that each species corresponded to a discrete clade, and that no strains were misclassified at the species level. Comparison between genotype and serovar was possible for 254 isolates. Of the 31 sequence types (STs) represented by at least two isolates, 18 STs included isolates assigned to two or three different serovars. Conversely, 14 serovars were identified that contained between 2 to 10 different STs. New observations were made on the global phylogeography of *Leptospira* spp., and the utility of MLST in making associations between human disease and specific maintenance hosts was demonstrated.

**Conclusion:**

The new MLST scheme, supported by an updated MLST website, allows the characterization and species assignment of isolates of the seven major pathogenic species associated with leptospirosis.

## Author Summary

Leptospirosis is a common zoonotic disease worldwide. Genotyping of the causative organisms provides important insights into disease transmission and informs preventive strategies and vaccine development. Multilocus sequence typing (MLST) is the most widespread genotyping methodology for bacterial pathogens, but the *Leptospira* scheme supported by a public MLST database is currently only applicable to *L. interrogans* and *L. kirschneri*. The purpose of this study was to extend the scheme to a total of seven pathogenic *Leptospira* species. This was achieved through the development of a modified scheme in which one of the seven MLST loci was replaced, together with newly designed primers for the remaining 6 loci. Comparison of the original and modified scheme demonstrated that they were very similar, hence sequence type (ST) assignments were largely carried over to the modified scheme. Phylogenetic trees reconstructed from concatenated sequences of the seven loci of the modified scheme demonstrated perfect classification of isolates into seven pathogenic species, which resided in clearly distinct phylogenetic clusters. Congruence was low between STs and serovars. The MLST scheme was used to gain new insights into the population genetic structure of *Leptospira* species associated with clinical disease and maintenance hosts in Asia.

## Introduction

Leptospirosis is a global zoonosis caused by pathogenic members of the *Leptospira* genus [1]–[5]. These are maintained by chronic carrier mammalian hosts (e.g. rats and dogs), and are typically transmitted to humans via water contaminated with urine or by direct contact with reservoir hosts [1]–[5]. Leptospirosis can be caused by 8 pathogenic *Leptospira* species [6]–[8], one of which (*L. alstonii*) is very rarely isolated with only two strains deposited in reference collections to date. A further five so-called ‘intermediate’ *Leptospira* species cause less severe disease [9]–[12], and there are six non-pathogenic species [6], [13], [14]. In addition, *L. kmetyi* clusters with the pathogenic species in a comparison of 16S rRNA gene sequence but has only been isolated from the environment thus far [8], and its ability to cause disease is uncertain. Three species (*L. interrogans*, *L. kirschneri* and *L. borgpetersenii*) cause the majority of human leptospirosis worldwide [5], [15]–[17], although clinical manifestations of leptospirosis caused by different pathogenic species are often indistinguishable. It is important, therefore, that typing methodologies incorporate all major pathogenic species, not least as a means of species identification [18], [19].

Multilocus sequence typing (MLST) is the genotyping method of choice for many bacterial pathogens, but the genetic divergence between species of pathogenic *Leptospira* [16] complicates the development of a single MLST scheme for all of the pathogenic species. The availability of whole genome sequence for *L. interrogans* facilitated the development by Thaipadungpanit *et al.* of a MLST scheme that was supported by a public MLST website and database for this species and the closely related *L. kirschneri* [20]. The primers used for this scheme worked variably for the other species and it was not recommended for use beyond *L. interrogans* and *L. kirschneri*. The subsequent publication of the whole genome sequence for *L. borgpetersenii* [16], and access to unpublished sequence data for other species including *L. weilii*, has provided the opportunity to now extend this scheme.

The objective of this study was to expand the *Leptospira* MLST scheme to the seven pathogenic species associated with the overwhelming majority of disease (*L. interrogans*, *L. kirschneri*, *L. borgpetersenii, L. noguchii, L. santarosai, L. weilii and L. alexanderi*).

## Methods

### Bacterial isolates, DNA extraction, species identification and serovar typing

The 327 *Leptospira* spp. used in this study are listed in Table S1. Genomic DNA was extracted as described previously [20], with the exception of isolates from the National Center for Emerging and Zoonotic Infectious Diseases, Centers for Diseases Control and Prevention, USA, which were extracted with the addition of dimethyl sulfoxide. For isolates for which the species was unknown, identification was undertaken by amplification and sequencing of the near full-length 16S rRNA gene (*rrs*), as described previously [20]. Sequence data for *rrs* has been deposited in GenBank (see Table S2 for accession numbers). The serovars of isolates from recent human leptospirosis cases in Thailand, Laos and Sri Lanka were determined by the WHO/FAO/OIE Collaborating Center for Reference and Research on Leptospirosis, Queensland Health Forensic and Scientific Services, Brisbane, Australia using the cross-agglutinin absorption test (CAAT). The serovars of contemporary isolates from China were determined using CAAT at the China Medical Culture Collection of National Institutes for Food and Drug Control, China.

Ethical approvals to obtain contemporary clinical isolates of *Leptospira* spp. are as follows. Isolates from patients in northeast Thailand between October 2000 and December 2006 were obtained during a prospective study of acute febrile illness [20], which was approved by the Ethical Committee of the Ministry of Public Health, Royal Government of Thailand. Isolates from patients in Laos between August 2006 and October 2009 were obtained during a prospective fever study approved by the Oxford Tropical Research Ethics Committee (OXTREC) and the National Ethics Committee for Health Research (NECHR), Ministry of Health, Government of Lao PDR. Isolates obtained from patients in Sri Lanka between June 2006 and May 2007 were obtained during a prospective fever study approved by the Research Ethics Committee of the Liverpool School of Tropical Medicine, UK and the Ethical Review Committee of the Faculty of Medicine of the University of Kelaniya, Sri Lanka. Written informed consent was obtained from all subjects except where not required (not required for data analyzed anonymously in Laos PDR at the time of the study). Other clinical isolates were obtained from the China Medical Culture Collection of National Institutes for Food and Drug Control, China, and the National Center for Emerging and Zoonotic Infectious Diseases, Centers for Diseases Control and Prevention, USA. All data were analyzed anonymously.

### Original MLST scheme

Sequence types and the associated sequence data for the seven MLST loci were downloaded from the *Leptospira* MLST website (http://leptospira.mlst.net) for 199 isolates. MLST was performed using the original scheme for an additional 52 *Leptospira* isolates that had not been genotyped previously. These included 38 *L. interrogans* isolates from Laos (n = 11), Malaysia (n = 2), Sri Lanka (n = 14), Japan (n = 1) the Philippines (n = 2), or China (n = 8), and 14 *L. kirschneri* isolates from Japan (n = 2) or reference collections (n = 12). Alleles were assigned and sequence types defined using the MLST website. The 251 isolates typed by the original scheme are detailed in Table S1.

### Modified MLST scheme

Six of the seven original MLST loci (*glmU*, *pntA*, *sucA*, *tpiA*, *pfkB* and *mreA*) were retained in the modified scheme. New primers were designed for these six loci to take into account the known genetic variability at the primer binding sites of *L. interrogans*, *L. borgpetersenii* and *L. weilii*, but the regions of the genes used to define the alleles were unchanged. The new primers corresponded to regions of these genes that were conserved in five *Leptospira* spp. genome sequences, and were designed using PrimerSelect software (DNASTAR Inc., Wisconsin, USA). Four of these genomes were from the NCBI database (*L. interrogans* serovar Lai strain 56601 (NC_004342) [21] and serovar Copenhageni strain Fiocruz L1-130 (NC_005823) [22]; *L. borgpetersenii* serovar Hardjo-bovis strain L550 (NC_008508) and strain JB197 (NC_008510) [16]), and one unpublished *L. weilii* genome was acquired from the Wellcome Trust Sanger Institute (European Nucleotide Archive (ENA) accession number ERS002113). The seventh locus, *fadD*, was replaced with *caiB*, a gene present on chromosome I that encodes carnitine dehydratase belonging to the CoA transferase family III. The primers used for the new PCR scheme are shown in Table 1.

**Table 1:** MLST loci and primers.

Locus	Primer	Sequence (5′ to 3′)	MgCl_2_(mM)	PCR product (bp)	Location of MLST locus*	Size of MLST locus (bp)
*glmU*	glmU-F_M_	AGGATAAGGTCGCTGTGGTA	1.5	650	3784955-3784512	444
	glmU-R_M_	AGTTTTTTTCCGGAGTTTCT				
*pntA*	pntA-F_M_	TAGGAAARATGAAACCRGGAAC	1.5	621	56347-56871	525
	pntA-R_M_	AAGAAGCAAGATCCACAAYTAC				
*sucA*	sucA-F_M_	TCATTCCACTTYTAGATACGAT	2.5	640	1227474-1227920	447
	sucA-R_M_	TCTTTTTTGAATTTTTGACG				
*tpiA*	tpiA-F_M_	TTGCAGGAAACTGGAAAATGAAT	3.5	639	1694673-1694248	426
	tpiA-R_M_	GTTTTACRGAACCHCCGTAGAGAAT				
*pfkB*	pfkB-F_M_	CGGAGAGTTTTATAARAAGGACAT	1.5	588	1386553-1386984	432
	pfkB-R_M_	AGAACACCCGCCGCAAAACAAT				
*mreA*	mreA-F_M_	GGCTCGCTCTYGACGGAAA	2.0	719	2734550-2734116	435
	mreA-R_M_	TCCRTAACTCATAAAMGACAAAGG				
*caiB*	caiB-F	CAACTTGCGGAYATAGGAGGAG	1.5	650	1562845-1563246	402
	caiB-R	ATTATGTTCCCCGTGAYTCG				

* Based on the published genome of *L. interrogans* serovar Lai strain 56601 (NC_004342). The subscript M denotes ‘modified’ and is used for those primers that were modified from the original MLST scheme. This does not apply to *caiB* which was used for the first time in the modified scheme.

Amplification and sequencing of *caiB* was performed for the 251 isolates genotyped by the original MLST scheme. All seven loci were sequenced for a further 74 isolates belonging to species that cannot be genotyped using the original scheme: *L. alexanderi* (n = 5), *L. borgpetersenii* (n = 34), *L. noguchii* (n = 9), *L. santarosai* (n = 10), and *L. weilii* (n = 16) (Table S1). Twelve of these were from recent cases of human leptospirosis in Laos (*L. borgpetersenii* [n = 2], *L. weilii* [n = 4]), Thailand (*L. borgpetersenii* [n = 4]) or Sri Lanka (*L. borgpetersenii* [n = 2]); and six were *L. borgpetersenii* isolates from recently trapped rodents in Japan. In addition, two *L. borgpetersenii* isolates were included based on *in silico* MLST using whole genome sequences from NCBI database (NC_008508 and NC_008510).

PCR reactions were performed in a volume of 25 µl containing 1.5–3.5 mM MgCl_2_ (Table 1), 200 µM dNTP (Roche, USA), 1.25 unit *Taq* DNA Polymerase (Roche, USA), 5 pmol of each forward and reverse primer (Table 1), and approximately 50 ng of *Leptospira* DNA. Amplifications were performed with the following conditions: one cycle of 95°C for 2 minutes, 30 cycles of 95°C for 10 seconds, 46°C for 15 seconds, and 72°C for 30 seconds, followed by a final period of 72°C for 7 minutes. PCR products were sequenced by Macrogen Inc. (Seoul, Korea), the sequences trimmed to the correct length (Table 1), and edited using SeqMan software (DNASTAR Inc., USA). Allele numbers were assigned to each unique *caiB* locus, together with new alleles for novel sequences at the six other loci. Allelic profiles (in the order *glmU-pntA-sucA-tpiA-pfkB-mreA-caiB*) were used to assign sequence types (STs) to all isolates. Wherever possible, the original ST number for a given isolate was preserved in the modified scheme.

### Nucleotide sequence analysis

Sequence alignment, the identification of polymorphic sites, and the construction of phylogenetic trees were performed using MEGA version 5.0 [23]. The rate of substitution at synonymous and non-synonymous sites (dN and dS, respectively) was determined using a modified Nei-Gojobori method [24]. Neighbor-joining trees were constructed from concatenated sequences of MLST loci using Kimura's two-parameter model. Maximum likelihood trees were constructed from concatenated sequences of MLST loci using an algorithm implemented in PhyML version 3.0.1 [25]. The model of sequence evolution used was the generalized time-reversible (GTR) model with gamma-distributed rate variation. The model parameters were adjusted as follows: transition/transversion ratio was fixed to 4.0, and the gamma shape parameter accounting for rate variation among sites and proportion of invariant sites was optimized. We set the program to search the tree using the Nearest Neighbor Interchange (NNI) method and chose BioNJ as an initial tree [25]. The MEGA program [23] was used to display and edit the tree. Discriminatory ability (D value) and 95% confidence intervals (CI) were estimated as described previously [26], [27]. Relatedness between STs was analyzed based on allelic profiles using eBURST version 3 [28], [29]. Neighbor-joining and maximum likelihood trees were also constructed for 100 isolates using the near full length *rrs* gene corresponding to positions 2417657 to 2418837 in the genome of *L. interrogans* serovar Lai strain 56601 (GenBank accession number NC_004342.2).

## Results

### MLST of seven pathogenic Leptospira species

The modified MLST scheme was applied to 327 isolates representing seven pathogenic *Leptospira* spp. (Table S1). A total of 190 different STs were resolved. The data for these can be accessed at the modified MLST website (http://leptospira.mlst.net/). The discriminatory ability for different species ranged from 0.5 ST per isolate for *L. interrogans* and *L. borgpetersenii* to 1.0 ST per isolate for *L. alexanderi*, *L. noguchii* and *L. santarosai* (Table 2). The number of alleles per locus ranged from 51 (*caiB*) to 70 (*pfkB*) (Table 2). Two *L. borgpetersenii* isolates from mice trapped in Japan, both of which were assigned as ST 197, contained a non-standard length allele (*caiB* 51) due to a 78 bp deletion (position 13–90) in *caiB*. We excluded these two isolates from the phylogenetic analysis. The species of the remaining 325 isolates were as follows: *L. interrogans* (n = 222), *L. borgpetersenii* (n = 34), *L. kirschneri* (n = 29), *L. weilii* (n = 16), *L. santarosai* (n = 10), *L. nogouchii* (n = 9), *L. alexanderi* (n = 5). The dN/dS ratio for each locus was less than one for all seven species, indicating a lack of positive selection (Table S3).

**Table 2:** Number of alleles and sequence types (STs) for *Leptospira* species.

Species	No. of isolates	No. of unique alleles at each locus							No. of STs	No. of STs per isolate
		*glmU*	*pntA*	*sucA*	*tpiA*	*pfkB*	*mreA*	*caiB*		
*L. alexanderi*	5	2	2	1	2	1	4	1	5	1.0
*L. borgpetersenii*	36	5	7	12	5	6	4	9	17	0.5
*L. interrogans*	222	12	19	14	24	32	18	16	111	0.5
*L. kirschneri*	29	13	12	10	7	6	7	8	23	0.8
*L. noguchii*	9	8	8	8	8	8	8	8	9	1.0
*L. santarosai*	10	6	8	8	4	8	7	3	10	1.0
*L. weilii*	16	6	8	7	5	9	7	6	15	0.9
**Total**	**327**	**52**	**64**	**59** *	**55**	**70**	**55**	**51**	**190**	**0.6**

* Allele 1 of *sucA* was shared between *L. interrogans* and *L. kirschneri.*

**Figure 1:**
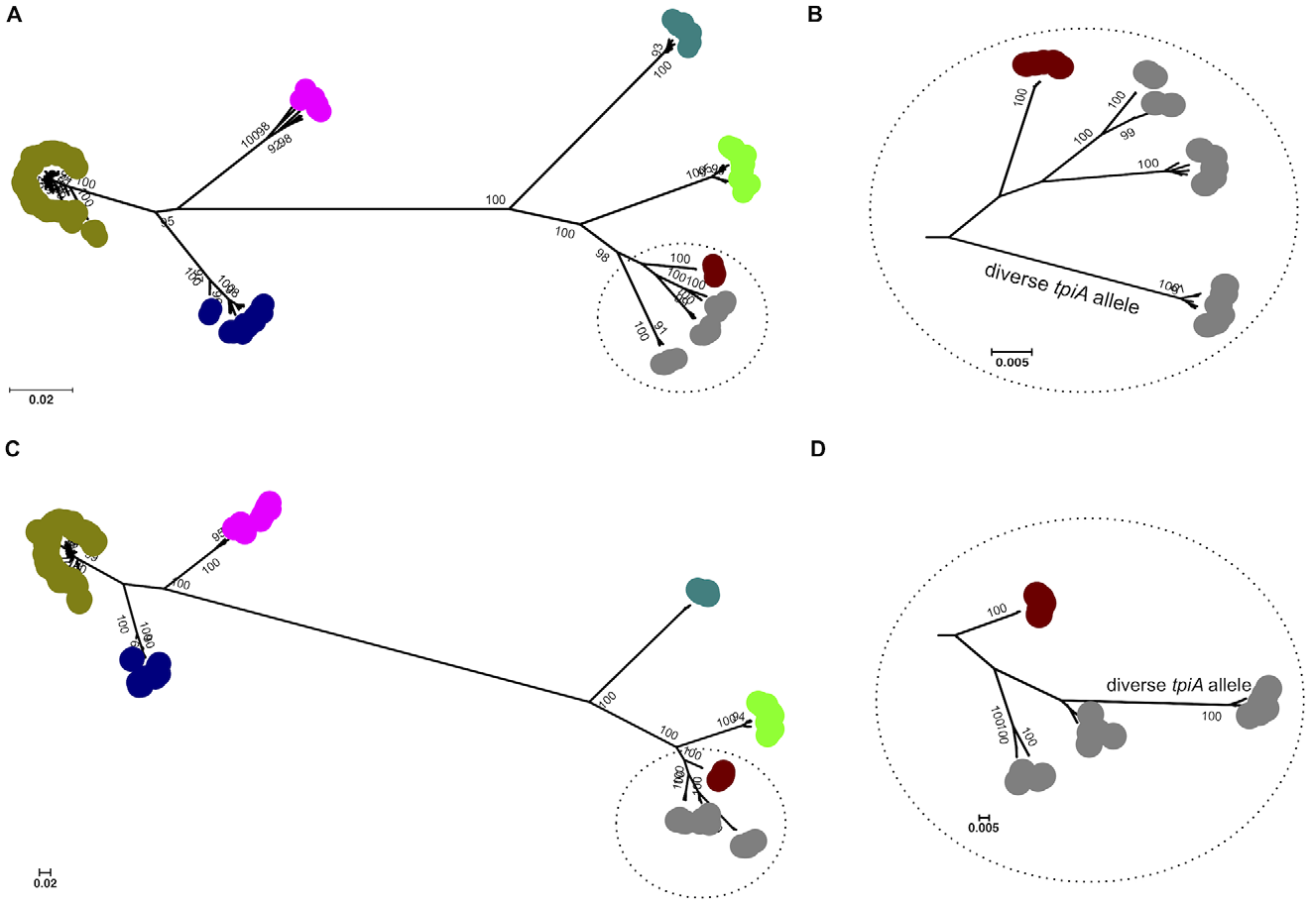
Phylogenetic trees based on concatenated sequences of 7 MLST loci. Phylogenetic trees were constructed based on concatenated sequences of 7 housekeeping loci for 189 unique STs using neighbor-joining (A) and maximum likelihood (C) methods. Both trees show six distinct clusters for 7 pathogenic *Leptospira* species. Relatedness between *L. weilii* isolates differ in the two trees; in the neighbor-joining tree, a cluster of *L. weilii* isolates with a highly diverse *tpiA* allele (*tpiA* 51) was distantly related to other *L. weilii* isolates, as shown in the enlarged view (B). The maximum likelihood tree demonstrated that *L. weilii* isolates fell into three sub-groups that clustered together, as shown in the enlarged view (D). Color code: khaki, *L. interrogans*; dark blue, *L. kirschneri*; pink, *L. nogouchii*; dark green, *L. santarosai*; light green, *L. borgpetersenii*; brown, *L. alexanderi*; grey, *L. weilli*. Only bootstrap support values over 90% are shown.

**Figure 2:**
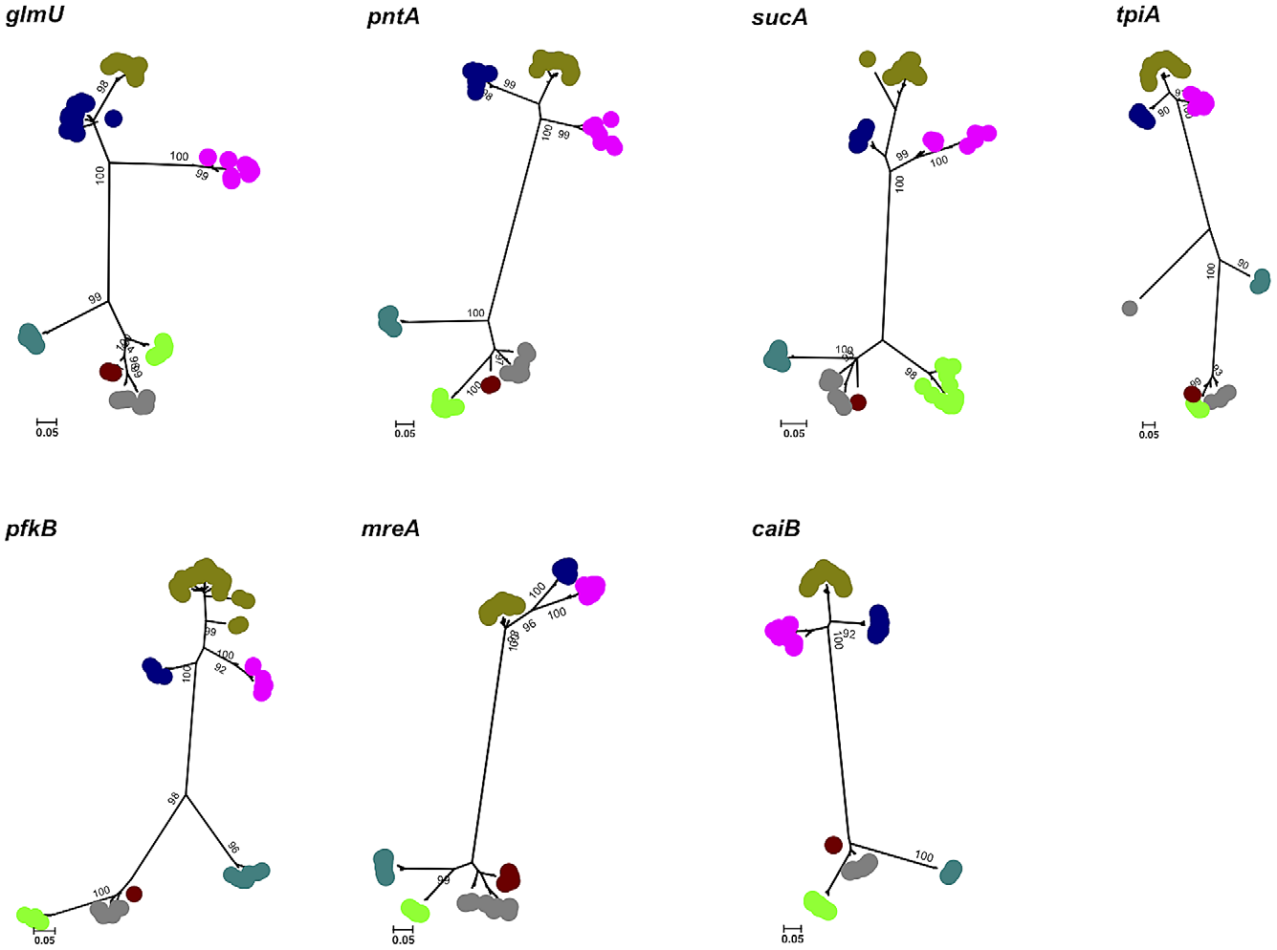
Maximum likelihood trees for each of seven MLST loci. The *tpiA* tree demonstrated a subdivision of *L. weilii* that was distinct and genetically distant from other *L. weilii* strains, as well as from *L. alexanderi* and *L. borgpetersenii*. This outlying group contained seven *L. weilii* isolates with a highly divergent *tpiA* (allele 51). Color code: khaki, *L. interrogans*; dark blue, *L. kirschneri*; pink, *L. nogouchii*; dark green, *L. santarosai*; light green, *L. borgpetersenii*; brown, *L. alexanderi*; grey, *L. weilli*.

Neighbor-joining and maximum-likelihood trees were constructed from concatenated sequences of the seven loci for 325 isolates. In general, phylogenetic analysis of the seven concatenated genes strongly supported current species assignments, although there was significant subdivision within the *L. weilii* population combined with a close relationship between *L. weilii* and *L. alexanderi* (Figure 1A–D). This was further explored in an analysis that considered the contribution of each individual locus to the phylogeny. Seven maximum likelihood trees were constructed, one for each of the seven MLST loci (Figure 2). All seven trees confirmed the close genetic relatedness between *L. weilii* and *L. alexanderi*. The *tpiA* tree was notable for a subdivision of *L. weilii* that was distinct and genetically distant from other *L. weilii* strains as well as from *L. alexanderi* and *L. borgpetersenii*. This outlying group contained seven *L. weilii* isolates with a highly divergent *tpiA* (allele 51). Constructing a tree using the concatenated sequences of all loci except *tpiA* resulted in all of the *L. weilii* isolates falling into only two lineages, and resolved the apparently polyphyletic nature of this species (Figure S1).

**Figure 3:**
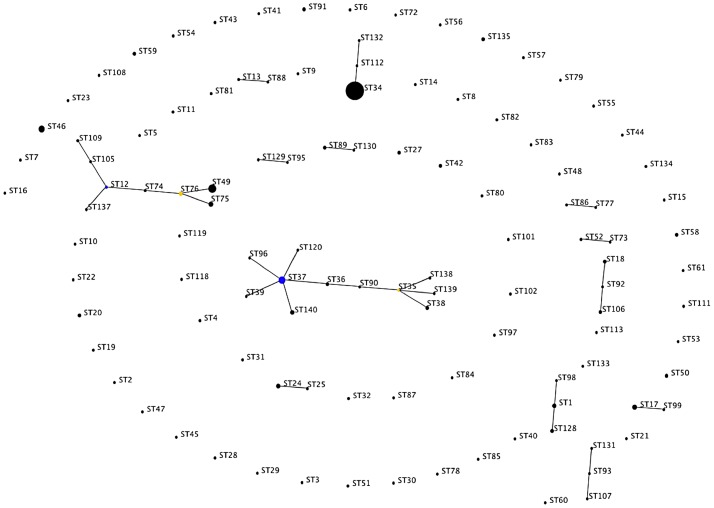
Analysis of *L. interrogans* using eBURST. eBURST version 3 was used to analyse the 111 unique STs resolved for all 222 *L. interrogans* isolates. Two main clonal complexes (CC) were defined (CC 12 and CC 37), together with predicted primary (blue) and sub-group (yellow) founders. The size of each dot is proportional to the number of isolates included in the analysis for each ST.

eBURST was used to identify groups of related STs (clonal complexes, CCs) among the 111 STs of *L. interrogans* (there were too few STs for the other species to use this procedure). This demonstrated two CCs, the putative founders of which were ST 37 and ST 12 respectively, and are thus named CC 37 and CC 12 (Figure 3). CC 37 contained 11 STs representing 22 isolates that originated from a wide geographic region including Thailand, Laos, Sri Lanka, China, Indonesia, Japan, Australia, Brazil, Jamaica and the Netherlands. CC 12 contained 8 STs representing 23 isolates from Asia (Sri Lanka, China, Malaysia and Thailand). Isolates belonging to the same CCs clustered together in the same branch of the phylogenetic tree (Figure S2).

### Comparison of original and modified MLST schemes

The impact of changing the single locus in the MLST scheme was evaluated by comparing the results of the original and modified schemes for the 251 isolates of *L. interrogans* and *L. kirschneri* in the study collection (n = 222 and n = 29, respectively). This comparison included almost all (117/120) unique STs registered previously in the MLST database based on the original scheme. The remaining three STs (STs 26, 33 and 114) were not included in this study because DNA or isolates were not available for the four associated isolates.

A neighbor-joining tree constructed from the concatenated sequences of the seven loci of the original scheme demonstrated two distinct phylogenetic clusters corresponding to *L. interrogans* and *L. kirschneri*, as reported previously [20]. Isolates belonging to the *L. interrogans* cluster were resolved into 111 STs (0.5 ST per isolate), and those in the *L. kirschneri* cluster were resolved into 22 STs (0.8 ST per isolate). Comparison of neighbor-joining trees for the two schemes reconstructed from concatenated sequences of seven loci showed these to be highly similar, retaining the clear separation of the two species. The modified scheme resolved five new STs (137, 138, 139, 140, and 141) as a result of sub-division of existing STs, while four original STs (94, 100, 103, 104) were lost through merging of STs (Table S1). The discriminatory power (D value) of the two schemes was not significantly different (original scheme 0.936 (95% CI 0.911–0.961) versus modified scheme 0.937 (95% CI 0.912–0.962). The D value of *caiB* was significantly higher than that of *fadD*, the allele which it replaced (*fadD*, 0.793 (95% CI 0.760–0.826); *caiB*, 0.876 (95% CI 0.852–0.901; p<0.05)). None of the *caiB* alleles were shared between *L. interrogans* and *L. kirschneri*.

### Comparison of phylogenies based on MLST versus 16S rRNA

**Figure 4:**
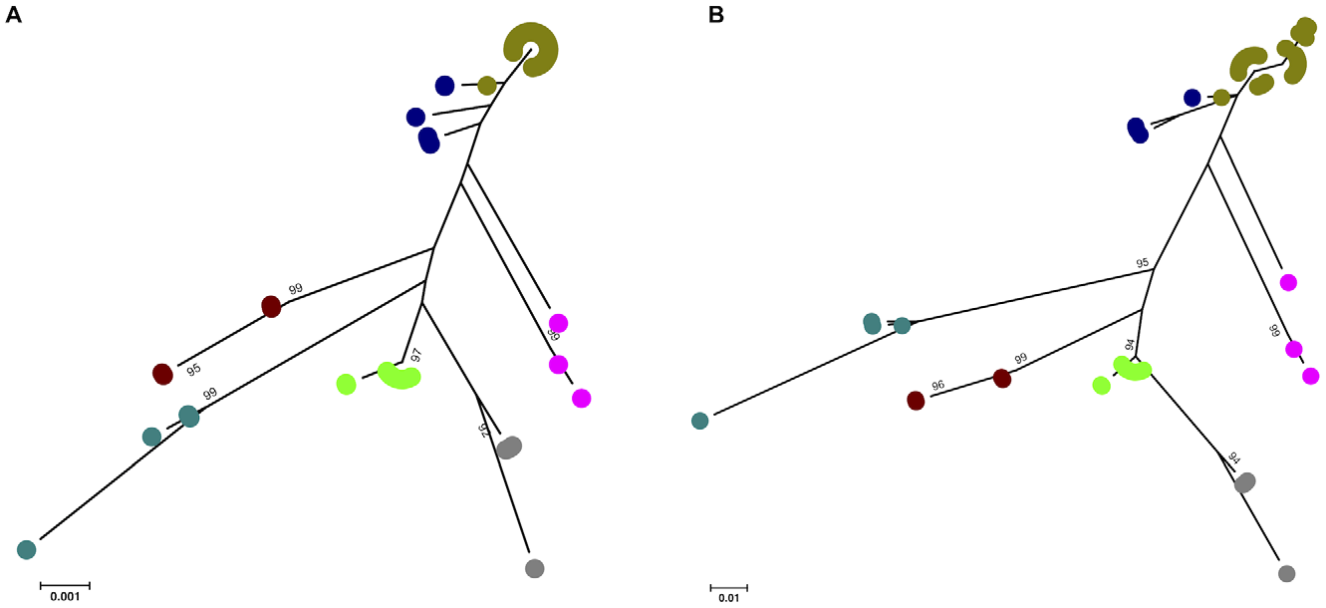
Phylogenetic trees based on the near full-length *rrs* gene. Neighbor-joining (A) and maximum likelihood (B) trees were constructed using the near full-length *rrs* gene of 100 isolates representing 7 pathogenic *Leptospira* species. Two isolates of *L. kirschneri* were misclassified as *L. interrogans* by *rrs*. Note the diversity of *rrs* sequences within *L. noguchii* species. Color code: khaki, *L. interrogans*; dark blue, *L. kirschneri*; pink, *L. nogouchii*; dark green, *L. santarosai*; light green, *L. borgpetersenii*; brown, *L. alexanderi*; grey, *L. weilli*.

Phylogenetic analysis has been performed previously for the *Leptospira* genus based on the *rrs* gene. We compared the phylogeny between this and MLST for the seven pathogenic *Leptospira* species included in this study. Sequence of the near-full length *rrs* gene was available for 100 study isolates, including 83 clinical isolates for which *rrs* sequencing was performed by us, and 17 sequences that were obtained from GenBank. Of these, ninety-four isolates had been genotyped by MLST and the remaining six isolates (*L. santarosai* or *L. noguchii*) were not represented in our collection. Neighbor-joining and maximum-likelihood trees based on *rrs* are shown in Figures 4A and 4B. The broad phylogenetic structure was similar to that observed using MLST, although several differences were noted. The clustering of *L. alexanderi* and *L. weilii* observed by MLST was not replicated by *rrs*, which assigned the two species to discrete branches. Three contemporary isolates had an ambiguous phylogeny by *rrs* that was not observed by MLST, as follows. One human isolate (ST 18) from Thailand in the *L. interrogans* cluster and two isolates (both ST 136) from shrews in Japan residing in the *L. kirschneri* cluster based on MLST were clustered by *rrs* into a single branch located between *L. interrogans* and *L. kirschneri*. This result suggests that recombination may occur in *rrs* between the two closely related *L. interrogans* and *L. kirschneri* species. Nucleotide distances between species were compared for data derived from MLST and *rrs*. These ranged from 3.35% to 18.43% for MLST (Table 3), and 0.15% to 1.01% for *rrs* (Table 4). The nucleotide distance between *L. alexanderi* and *L. weilii* was 3.35% by MLST, but only 0.75% by *rrs*.

**Table 3:** Average (range) nucleotide distance based on concatenated sequences of 7 MLST loci.

	*L. interrogans*	*L. kirschneri*	*L. noguchii*	*L. santarosai*	*L. borgpetersenii*	*L. alexanderi*
*L. kirschneri*	6.59%					
	(4.9%–7.53%)					
*L. noguchii*	7.34%	7.34%				
	(5.37%–10.77%)	(5.21%–9.93%)				
*L. santarosai*	18.43%	18.1%	18.1%			
	(15.45%–23.51%)	(14.75%–22.6%)	(14.73%–22.53%)			
*L. borgpetersenii*	18.39%	17.85%	18.09%	12.59%		
	(14.99%–25.47%)	(12.99%–24.7%)	(13.63%–26.02%)	(9.09%–20.13%)		
*L. alexanderi*	17.92%	17.61%	17.98%	11.81%	6.43%	
	(15.35%–26.1%)	(14.75%–26.25%)	(14.39%–27.15%)	(7.36%–20.34%)	(3.45%–8.8%)	
*L. weilii*	16.8%	16.51%	16.75%	10.75%	6.82%	3.35%
	(14.62%–19.38%)	(13.84%–19.67%)	(13.61%–20.41%)	(5.4%–15.2%)	(4.21%–7.86%)	(2.71%–3.28%)

**Table 4:** Average nucleotide distance based on the near-full length *rrs* gene*.

	*L. interrogans*	*L. kirschneri*	*L. noguchii*	*L. santarosai*	*L. borgpetersenii*	*L. alexanderi*
*L. kirschneri*	0.15%					
*L. noguchii*	0.41%	0.30%				
*L. santarosai*	1.01%	0.90%	0.70%			
*L. borgpetersenii*	0.67%	0.56%	0.58%	0.68%		
*L. alexanderi*	0.80%	0.54%	0.70%	1.00%	0.55%	
*L. weilii*	0.81%	0.78%	0.72%	0.87%	0.40%	0.75%

* Based on 100 sequences of a region of the *rrs* gene (NC_004342.2) from position 2417657 to 2418837 on the complementary strand.

### Congruence between STs and serovar designations

Typing of *Leptospira* spp. based on serovars is a long established approach for epidemiological surveillance. We examined whether isolates of the same serovar were also closely related by genotype, and *vice versa*. This was possible for 254/325 isolates that had both a ST and serovar designation, the remaining 71 isolates being of unknown serovar. We first considered the serovars corresponding to the 31 STs represented by at least two isolates. Of these, 13 STs contained isolates belonging to a single serovar and 18 STs contained isolates that were assigned to two or three different serovars (Table S4). We extended this analysis to those isolates belonging to *L. interrogans* CC 37 or CC 12. The 23 isolates in CC 12 belonged to three different serovars (Pyrogenes, Bangkok and Fugis), and the 22 isolates in CC 37 belonged to nine serovars (Canicola, Gem, Guaratuba, Medanensis, Paidjan, Portlandvere, Pyrogenes, Pomona, and Hebdomadis). We then identified those serovars that contained more than one ST. This was the case for 16 serovars, the number of STs per serovar ranging from 2 to 10 (Table S5). There was a variable degree of relatedness between STs belonging to the same serovar. These observations indicate that serovar is a poor indicator of genetic relatedness and can vary within a given clone or lineage, an observation that is most likely to be due to horizontal gene transfer.

### MLST phylogeny and geographical distribution of seven pathogenic Leptospira species

The *L. interrogans* cluster contained four sub-groups, the largest of which contained 104 different STs comprising isolates with a global distribution. The other three sub-groups contained isolates from the Asia-Pacific region comprising eight STs (Figure S2). The *L. kirschneri* cluster contained two major sub-groups, the larger of which contained reference isolates originating from Asia, Europe, Africa, North America and South America. The smaller sub-group contained ST 68 (n = 2) and ST 71 (n = 1), all of which were clinical isolates from Thailand. These three isolates were distinct in that they contained a *sucA* allele (allele 1) that is also found in *L. interrogans*, the only example of shared alleles between the two species. The *L. noguchii* cluster containing nine reference isolates were divided into three sub-groups that originated from United States and Central America, Hawaii, and South America, respectively. The *L. borgpetersenii* cluster contained two sub-groups, the larger of which contained reference isolates from Europe, Asia, and Africa, and recent isolates from human leptospirosis cases in Thailand, Laos and Sri Lanka, and rodents in Japan (n = 6). The smaller cluster contained only reference isolates originating from several continents but not Asia. The *L. santarosai* cluster contained 10 reference isolates each of a different ST, nine of which were from Central and South America. *L. weilii* fell into three sub-groups. The first contained reference isolates from China and Indonesia and recent clinical isolates from Laos; the second contained reference isolates from China, Vietnam and Malaysia plus a recent clinical isolate from Laos; and the third contained 4 reference isolates from Australia, Indonesia and Malaysia. All members the first sub-group contained the highly divergent *tpiA* (allele 51). A single *L. alexanderi* group contained reference isolates originating from China.

### Observations arising from genotyping of contemporary Asian isolates

MLST was performed on 126 new isolates, including contemporary isolates from humans or animals from Laos, Malaysia, the Philippines, Sri Lanka, Japan and China. This allowed us to examine the population of *Leptospira* across a wider region of Asia than has been performed previously. We noted that the dominant clone defined previously in Thailand (*L. interrogans* ST 34) [20] was also isolated from humans in neighboring Laos (8/27 [30%] of isolates tested). The closest relative of ST 34 found in this study was the single locus variant ST 112 (Figure 3), which was isolated from a human in India. Most of the *L. borgpetersenii* isolates associated with human leptospirosis in Thailand, Laos, and Sri Lanka were closely related, with seven out of the eight isolates belonging to ST 143 (n = 4) or ST 144 (n = 3), which are single locus variants. ST 143 was also represented in recent isolates from rats in Japan (n = 3), as well as reference isolates from China (n = 1) and Indonesia (n = 1). These observations indicate that this *L. borgpetersenii* lineage is widely distributed in Asia, and that the rat is one of the maintenance hosts in this region. A reference isolate from a case of human leptospirosis originating in Sri Lanka in 1964 was also *L. borgpetersenii* ST 144, indicating the presence of this lineage in Asia for at least half a century. *L. borgpetersenii* ST 146 was recovered from shrews in Japan in 2011, and a shrew in Czechoslovakia in 1953. These data demonstrate the potential of MLST to define the phylogeography of *Leptospira* species over time, and to create links with their maintenance host. *L. weilii* formed a small minority of recent clinical isolates from cases of human leptospirosis in Asia (4/140 [3%]).

### Phylogeny of Leptospira spp. isolated from humans and animals

The discrimination provided by MLST allowed us to re-examine the relationship between isolates that are pathogenic for man and their putative maintenance host. For this we made the assumption that the same ST isolated from an animal and a human implied that the strain had crossed the species barrier. This analysis was possible for 316 isolates of known origin, the remainder being of unknown origin (n = 8) or from the environment (n = 1). The 316 isolates originated from humans (n = 230), rodents (n = 48), amphibians (n = 2), and other mammals (n = 36). Several STs contained isolates obtained both from humans and rodents (n = 10), humans and other mammals (n = 4), or humans, rodents and other mammals (n = 1). We found a single instance where an isolate of *L. kirschneri* ST 122 associated with human leptospirosis was maintained in a rodent. Our analysis provided confirmatory evidence for the role of rodents as major maintenance hosts for *L. interrogans*, and bovines as major maintenance hosts for *L. borgpetersenii* and *L. weilii*. This also demonstrates the potential utility of MLST for the investigation of maintenance hosts during outbreak and other epidemiological investigations.

## Discussion

This study has described the successful modification of a *Leptospira* MLST scheme to allow the characterization of isolates of seven pathogenic *Leptospira* species. The original and modified schemes provide similar levels of resolution, and the modified scheme presents considerable advantages for the scientific community with minimal negative impact. We contacted all of the investigators who had deposited MLST data into the public database, and were able to include all but 4 isolates (old STs 26, 33 and 114) in the modified scheme. The current MLST website and database (http://leptospira.mlst.net/) has been updated to reflect the new improved scheme, and the old scheme will remain available via the website for a period of 6 months and then removed to avoid confusion.

The modified MLST scheme assigned isolates to distinct clades with 100% accuracy and can, therefore, be used to assign both species and genotype to isolates of the seven *Leptospira* species tested here. Furthermore, we were able to identify relatedness between STs in specific geographic regions. For example, a clonal complex of *L. interrogans* (CC 12) was identified that contained recent isolates from several countries in southeast Asia, and genetic relatedness was identified between isolates from Australia and Papua New Guinea. Although the number of *Leptospira* isolates that have undergone MLST is relatively small to date, this suggests that the scheme is capable of describing the molecular epidemiology of the main pathogenic species within the genus on a global scale. There is notable genetic diversity both within a given species and between species, indicating that MLST would also be predicted to be capable of demonstrating transmission pathways of specific clones between maintenance hosts and man.

Several other multilocus sequence based typing schemes for *Leptospira* spp. have been reported in the literature. The most comparable scheme to the one described in our study is that described by Leon *et al.*, which is also based on seven housekeeping genes and is restricted to *L. interrogans* and *L. kirschneri* [30]. The species identity of 1/51 isolates tested was apparently misclassified by MLST, although misidentification of the isolate was also considered a possibility. This scheme is not supported by a public database and website and does not extend to other pathogenic species. A six locus scheme described by Ahmed *et al.* [31] included *rrs2* (one of two 16S rRNA genes that would be predicted to be highly conserved), three housekeeping genes, and two genes encoding surface expressed proteins (*lipL41* and *lipL32*), albeit that these appeared evolutionary neutral [32]. Discrepancy between serovar identity and speciation was evident for 2/120 isolates in its first description, with multiple examples of apparent serovar misclassification or mislabeling in a more extensive validation of the scheme using 271 isolates [33]. For example, the largest cluster of *L. interrogans* also contained three other *Leptospira* species [33]. This scheme is not associated with a public database and website although sequences for the 271 strains can be downloaded from GenBank, and phylogenetic analysis of new sequences using this scheme requires their download and offline analysis. A further scheme consisting of four loci including genes encoding surface expressed protein has been evaluated *in silico* for 38 pathogenic *Leptospira* strains, which demonstrated correct species assignment for 34 of these [34].

The finding in this study that serovar is a poor indicator of genetic relatedness and can vary within a given clone or lineage is most readily explained by horizontal gene transfer of genes encoding the surface determinants that confer serovar designation. Serovar variation is thought to relate to differences in the LPS O-antigen encoded by the *rfb* gene cluster, although the precise nature of this variation is not fully defined. A comparison of the sequence of the *rfb* cluster in five isolates all of different serovar (four *L. interrogans* and one *L. borgpetersenii*) demonstrated the presence of a set of genes that were present in all isolates, together with several genes that were variably present [21]. In addition, a study that used comparative genomic hybridization to compare the gene content of *L. interrogans* serovar Lai strain Lai with eleven *L. interrogans* belonging to different serovars reported notable divergence in the *rfb* cluster [35]. Large-scale whole genome sequencing projects that are currently underway are likely to shed important new evidence on the genetic structure and putative stability of the regions involved in serovar designation.

In summary, the modified MLST scheme described here proved highly discriminatory for seven pathogenic species of *Leptospira*, providing both isolate characterization and robust assignment to species in addition to phylogenetic evidence for the relatedness between the species. Crucially, this scheme is also supported by a public website. We recommend that this MLST scheme and the updated website at http://leptospira.mlst.net/ should be used for the future characterization of *Leptospira* isolates.

## Supporting Information

Figure S1 **Maximum likelihood tree based on concatenated sequences of 6 MLST loci, excluding** ***tpiA*** **.** All of the *L. weilii* isolates resided in only two lineages, resolving the apparently polyphyletic nature of this species. Color code: khaki, *L. interrogans*; dark blue, *L. kirschneri*; pink, *L. nogouchii*; dark green, *L. santarosai*; light green, *L. borgpetersenii*; brown, *L. alexanderi*; grey, *L. weilli*.
(PPTX)

Figure S2 **Phylogenetic tree of** ***L. interrogans*** **.** A neighbor-joining tree was constructed from concatenated sequences of the 7 MLST loci for 111 unique STs of *L. interrogans*. Colored triangles refer to clonal complexes (CC) as defined by eBURST. Red is for CC 37, and blue is for CC 12. The largest cluster contains isolates with a global distribution. The three smaller groups contain isolates that all originated from the Asia-Pacific region. ST 57 and its double locus variant ST 135 were isolated from rats in the Philippines, trapped in Manila and Los Baños (about 70 km apart) a period of 50 years apart (1957 and 2006/7, respectively). An ST representing an isolate from Papua New Guinea (ST 53) clustered with isolates from Australia (STs 52 and 73).
(TIFF)

Table S1 **List of** ***Leptospira*** **spp. used in this study.**
(DOC)

Table S2 **Accession numbers for** ***rrs*** **fragments sequenced in this study.**
(DOC)

Table S3 **dN/dS ratio of each MLST locus for 7** ***Leptospira*** **species.**
(DOC)

Table S4 **Serovars within individual sequence types (STs).**
(DOC)

Table S5 **Sequence types (STs) within individual serovars.**
(DOC)
