# Supplementary figures and images for: A Single Multilocus Sequence Typing (MLST) Scheme for Seven Pathogenic Leptospira Species
Source: PLoS Negl Trop Dis. 2013 Jan 24;7(1):e1954. doi: 10.1371/journal.pntd.0001954 (PMC3554523; doi:10.1371/journal.pntd.0001954)

## Slide 1
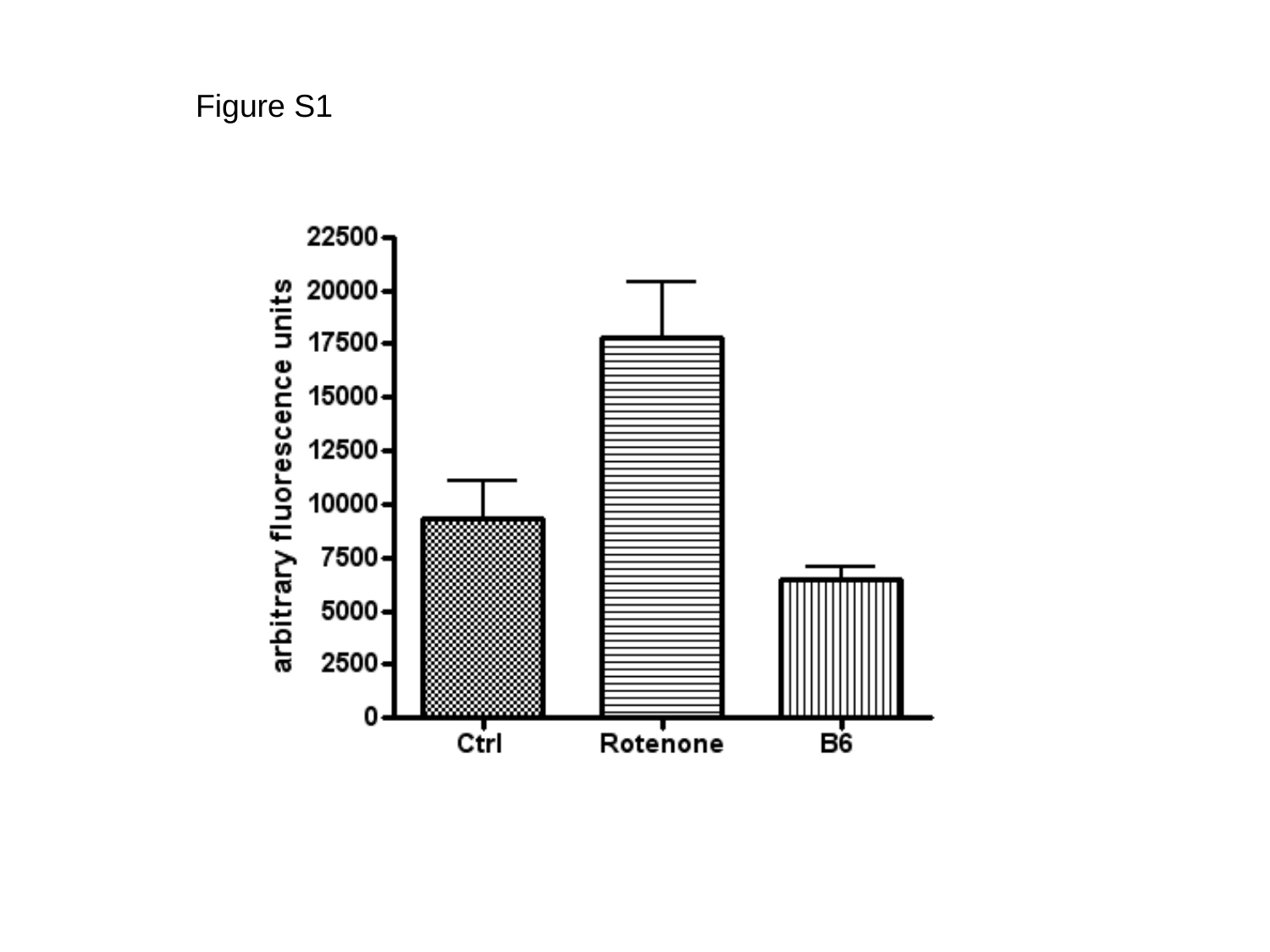

Figure S1

Supplement: Figure S1 — Maximum likelihood tree based on concatenated sequences of 6 MLST loci, excluding tpiA . All of the L. weilii isolates resided in only two lineages, resolving the apparently polyphyletic nature of this species. Color code: khaki, L. interrogans; dark blue, L. kirschneri; pink, L. nogouchii; dark green, L. santarosai; light green, L. borgpetersenii; brown, L. alexanderi; grey, L. weilli. (PPTX) [file pntd.0001954.s001.pptx]

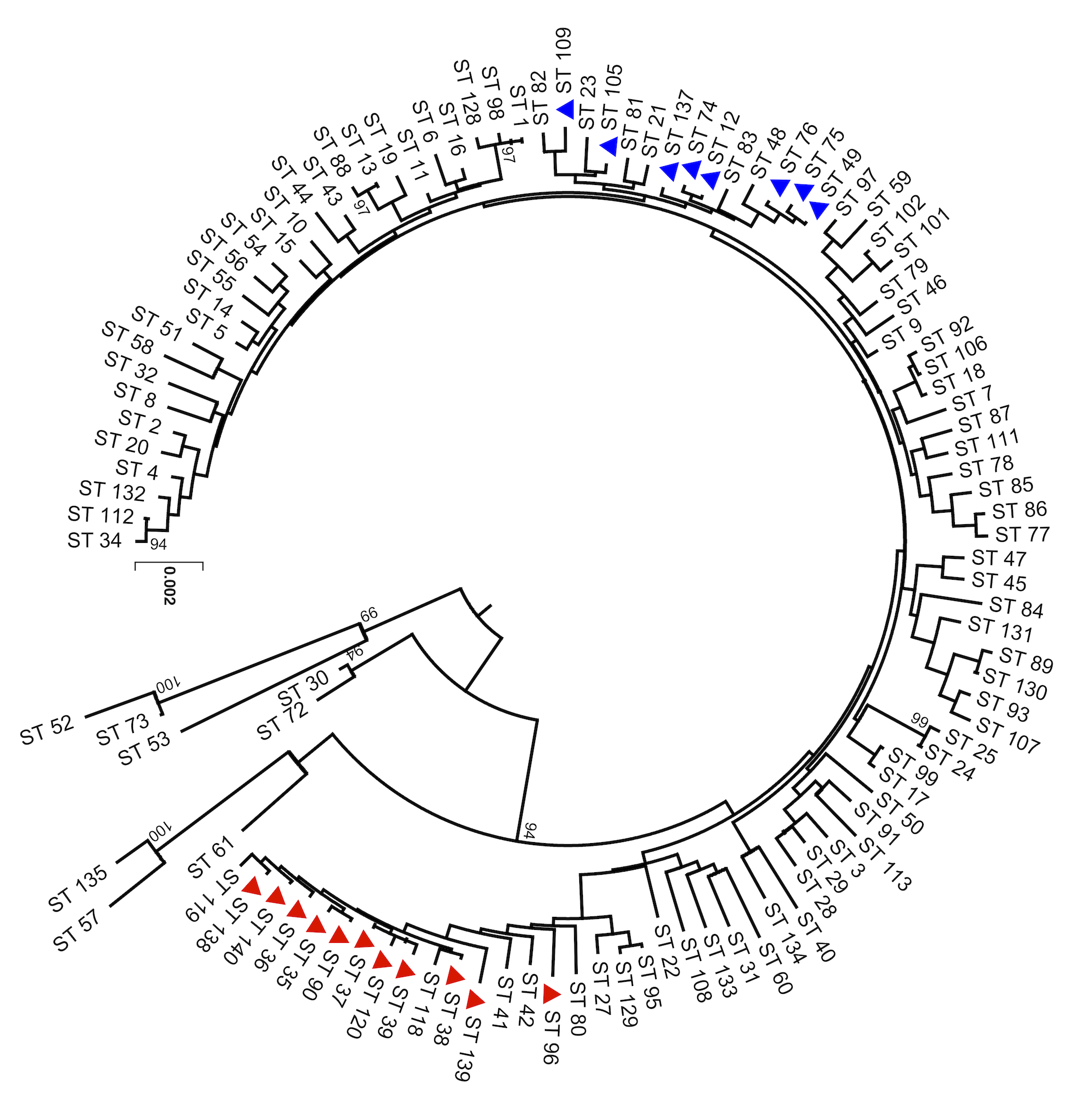

Supplement: Figure S2 — Phylogenetic tree of L. interrogans . A neighbor-joining tree was constructed from concatenated sequences of the 7 MLST loci for 111 unique STs of L. interrogans. Colored triangles refer to clonal complexes (CC) as defined by eBURST. Red is for CC 37, and blue is for CC 12. The largest cluster contains isolates with a global distribution. The three smaller groups contain isolates that all originated from the Asia-Pacific region. ST 57 and its double locus variant ST 135 were isolated from rats in the Philippines, trapped in Manila and Los Baños (about 70 km apart) a period of 50 years apart (1957 and 2006/7, respectively). An ST representing an isolate from Papua New Guinea (ST 53) clustered with isolates from Australia (STs 52 and 73). (TIFF) [file pntd.0001954.s002.tiff]
