# Supplementary material for: A Single Multilocus Sequence Typing (MLST) Scheme for Seven Pathogenic Leptospira Species
Source: PLoS Negl Trop Dis. 2013 Jan 24;7(1):e1954. doi: 10.1371/journal.pntd.0001954 (PMC3554523; doi:10.1371/journal.pntd.0001954)
Supplement: Table S1 — List of Leptospira spp. used in this study. (DOC) [file pntd.0001954.s003.doc]

**Table S1. List of *Leptospira* spp. used in this study.**

| **Species** | **Serovar** | **Strain** | **Country** | **Year** | **Host** | **Source^c^** | **Original ST^d^** | **Modified ST^e^** |
| --- | --- | --- | --- | --- | --- | --- | --- | --- |
| *L. interrogans^a^* | Lai | 56601 | China |  | Human | GenBank | 1 | 1 |
| *L. interrogans^a^* | Lai | Lai | China | 1958 | Human | WCCRRL | 1 | 1 |
| *L. interrogans* | Undesignated | H0703 | China | 2007 | Rattus norvegicus | NICDCP | 1 | 1 |
| *L. interrogans^a^* | Paidjan | Paidjan | Indonesia | 1939 | Human | WCCRRL | 2 | 2 |
| *L. interrogans^a^* | Schueffneri | Vleermuis 90 C | Indonesia | 1938 | Bat | WCCRRL | 3 | 3 |
| *L. interrogans^a^* | Mujunkumi | Yezsh 273 | Russia | 1966 | Long-eared hedgehog | WCCRRL | 4 | 4 |
| *L. interrogans^a^* | Mooris | Moores | Malaysia | 1953-1955 | Human | WCCRRL | 5 | 5 |
| *L. interrogans^a^* | Sentot | Sentot | Indonesia | 1937 | Human | WCCRRL | 6 | 6 |
| *L. interrogans^a^* | Sumneri | Sumner | Malaysia | 1957^b^ | Human | WCCRRL | 7 | 7 |
| *L. interrogans^a^* | Bindjei | Bindjei | Indonesia | 1930-1939 | Human | WCCRRL | 8 | 8 |
| *L. interrogans^a^* | Nanla | A 6 | China | 1962 | Human | WCCRRL | 9 | 9 |
| *L. interrogans^a^* | Undesignated | Heusden P2062 |  |  |  | NIH | 10 | 10 |
| *L. interrogans^a^* | Djasiman | Djasiman | Indonesia | 1938 | Human | WCCRRL | 11 | 11 |
| *L. interrogans^a^* | Bangkok | Bangkok D 92 | Thailand |  | Dog | NIH | 12 | 12 |
| *L. interrogans^a^* | Fugis | Fudge | Malaysia | 1957^b^ | Human | WCCRRL | 12 | 137 |
| *L. interrogans^a^* | Pyrogenes | Salinem | Indonesia | 1924 | Human | NIH | 13 | 13 |
| *L. interrogans^a^* | Biggis | Biggs | Malaysia | 1953-1955 | Human | WCCRRL | 14 | 14 |
| *L. interrogans^a^* | Abramis | Abraham | Malaysia | 1953-1955 | Human | WCCRRL | 15 | 15 |
| *L. interrogans^a^* | Benjamini | Benjamin | Indonesia | 1937 | Human | WCCRRL | 16 | 16 |
| *L. interrogans^a^* | Copenhageni | Fiocruz L1-130 | Brazil |  | Human | GenBank | 17 | 17 |
| *L. interrogans^a^* | Copenhageni | M 20 | Denmark | 1938 | Human | NIH | 17 | 17 |
| *L. interrogans^a^* | Icterohaemorrhagiae | Ictero No.1 | Japan | 1915^b^ | Human | NIH | 17 | 17 |
| *L. interrogans^a^* | Icterohaemorrhagiae | RGA | Belgium | 1915 | Human | WCCRRL | 17 | 17 |
| *L. interrogans^a^* | Grippotyphosa | L1006 | Thailand | 2002 | Human | MORU | 18 | 18 |
| *L. interrogans^a^* | Mankarso | Mankarso | Indonesia | 1938 | Human | WCCRRL | 19 | 19 |
| *L. interrogans^a^* | Haemolytica | Marsh | Malaysia | 1953-1955 | Human | WCCRRL | 20 | 20 |
| *L. interrogans^a^* | Hardjo | Hardjoprajitno | Indonesia | 1938 | Human | NIH | 20 | 20 |
| *L. interrogans^a^* | Smithi | Smith | Malaysia | 1953-1955 | Human | WCCRRL | 21 | 21 |
| *L. interrogans^a^* | Autumnalis | LP101 | Thailand | 2003 | Human | MORU | 22 | 22 |
| *L. interrogans^a^* | Naam | Naam | Indonesia | 1936 | Human | WCCRRL | 23 | 23 |
| *L. interrogans^a^* | Bratislava | Jez Bratislava | Czechoslovakia | 1953 | Hedgehog | NIH | 24 | 24 |
| *L. interrogans^a^* | Jalna | Jalna | Czechoslovakia | 1953 | Yellow-throat mouse | WCCRRL | 24 | 24 |
| *L. interrogans^a^* | Muenchen | München C 90 | Germany | 1942 | Human | WCCRRL | 24 | 24 |
| *L. interrogans^a^* | Lora | Lora | Italy | 1941 | Human | WCCRRL | 25 | 25 |
| *L. interrogans^a^* | Autumnalis | Akiyami A | Japan | 1922 | Human | WCCRRL | 27 | 27 |
| *L. interrogans^a^* | Undesignated | Aki A (NIJ) | Japan |  |  | WCCRRL | 27 | 27 |
| *L. interrogans^a^* | Birkini | Birkin | Malaysia | 1953-1955 | Human | WCCRRL | 28 | 28 |
| *L. interrogans^a^* | Bangkinang | Bangkinang 1 | Indonesia | 1929 | Human | WCCRRL | 29 | 29 |
| *L. interrogans^a^* | Robinsoni | Robinson | Australia | 1951 | Human | WCCRRL | 30 | 30 |
| *L. interrogans^a^* | Zanoni | Zanoni | Australia | 1933 | Human | WCCRRL | 31 | 31 |
| *L. interrogans^a^* | Evansi | 267-1348 | Malaysia | 1961-1966 | Water | WCCRRL | 32 | 32 |
| *L. interrogans^a^* | Autumnalis | L0013 | Thailand | 2000 | Human | MORU | 34 | 34 |
| *L. interrogans^a^* | Autumnalis | L0015 | Thailand | 2000 | Human | MORU | 34 | 34 |
| *L. interrogans^a^* | Autumnalis | L0020 | Thailand | 2000 | Human | MORU | 34 | 34 |
| *L. interrogans^a^* | Autumnalis | L0025 | Thailand | 2000 | Human | MORU | 34 | 34 |
| *L. interrogans^a^* | Autumnalis | L0088 | Thailand | 2000 | Human | MORU | 34 | 34 |
| *L. interrogans^a^* | Autumnalis | L0116 | Thailand | 2000 | Human | MORU | 34 | 34 |
| *L. interrogans^a^* | Autumnalis | L0133 | Thailand | 2000 | Human | MORU | 34 | 34 |
| *L. interrogans^a^* | Autumnalis | L0135 | Thailand | 2000 | Human | MORU | 34 | 34 |
| *L. interrogans^a^* | Autumnalis | L0382 | Thailand | 2001 | Human | MORU | 34 | 34 |
| *L. interrogans^a^* | Autumnalis | L0388 | Thailand | 2001 | Human | MORU | 34 | 34 |
| *L. interrogans^a^* | Autumnalis | L0431 | Thailand | 2001 | Human | MORU | 34 | 34 |
| *L. interrogans^a^* | Autumnalis | L0442 | Thailand | 2001 | Human | MORU | 34 | 34 |
| *L. interrogans^a^* | Autumnalis | L0453 | Thailand | 2001 | Human | MORU | 34 | 34 |
| *L. interrogans^a^* | Autumnalis | L0468 | Thailand | 2001 | Human | MORU | 34 | 34 |
| *L. interrogans^a^* | Autumnalis | L0474 | Thailand | 2001 | Human | MORU | 34 | 34 |
| *L. interrogans^a^* | Autumnalis | L0521 | Thailand | 2001 | Human | MORU | 34 | 34 |
| *L. interrogans^a^* | Autumnalis | L0528 | Thailand | 2001 | Human | MORU | 34 | 34 |
| *L. interrogans^a^* | Autumnalis | L0562 | Thailand | 2001 | Human | MORU | 34 | 34 |
| *L. interrogans^a^* | Autumnalis | L0587 | Thailand | 2001 | Human | MORU | 34 | 34 |
| *L. interrogans^a^* | Autumnalis | L0607 | Thailand | 2001 | Human | MORU | 34 | 34 |
| *L. interrogans^a^* | Autumnalis | L0643 | Thailand | 2001 | Human | MORU | 34 | 34 |
| *L. interrogans^a^* | Autumnalis | L0661 | Thailand | 2001 | Human | MORU | 34 | 34 |
| *L. interrogans^a^* | Autumnalis | L0752 | Thailand | 2002 | Human | MORU | 34 | 34 |
| *L. interrogans^a^* | Autumnalis | L0894 | Thailand | 2002 | Human | MORU | 34 | 34 |
| *L. interrogans^a^* | Autumnalis | L0984 | Thailand | 2002 | Human | MORU | 34 | 34 |
| *L. interrogans^a^* | Autumnalis | L1000 | Thailand | 2002 | Human | MORU | 34 | 34 |
| *L. interrogans^a^* | Autumnalis | L1059 | Thailand | 2002 | Human | MORU | 34 | 34 |
| *L. interrogans^a^* | Autumnalis | L1096 | Thailand | 2002 | Human | MORU | 34 | 34 |
| *L. interrogans^a^* | Autumnalis | L1118 | Thailand | 2002 | Human | MORU | 34 | 34 |
| *L. interrogans^a^* | Autumnalis | L1227 | Thailand | 2002 | Human | MORU | 34 | 34 |
| *L. interrogans^a^* | Autumnalis | L1229 | Thailand | 2002 | Human | MORU | 34 | 34 |
| *L. interrogans^a^* | Autumnalis | L1254 | Thailand | 2002 | Human | MORU | 34 | 34 |
| *L. interrogans^a^* | Autumnalis | LP085 | Thailand | 2003 | Human | MORU | 34 | 34 |
| *L. interrogans^a^* | Autumnalis | RY021 | Thailand | 2003 | Human | MORU | 34 | 34 |
| *L. interrogans^a^* | Autumnalis | UT104 | Thailand | 2003 | Human | MORU | 34 | 34 |
| *L. interrogans^a^* | Autumnalis | UT105 | Thailand | 2003 | Human | MORU | 34 | 34 |
| *L. interrogans^a^* | Autumnalis | UT108 | Thailand | 2003 | Human | MORU | 34 | 34 |
| *L. interrogans^a^* | Autumnalis | UT226 | Thailand | 2004 | Human | MORU | 34 | 34 |
| *L. interrogans^a^* | Autumnalis | UT227 | Thailand | 2004 | Human | MORU | 34 | 34 |
| *L. interrogans^a^* | Autumnalis | UT285 | Thailand | 2004 | Human | MORU | 34 | 34 |
| *L. interrogans^a^* | Autumnalis | UT342 | Thailand | 2005 | Human | MORU | 34 | 34 |
| *L. interrogans^a^* | Autumnalis | UT567 | Thailand | 2006 | Human | MORU | 34 | 34 |
| *L. interrogans^a^* | Autumnalis | UT670 | Thailand | 2006 | Human | MORU | 34 | 34 |
| *L. interrogans^a^* | Autumnalis | YT0012 | Thailand | 2003 | Human | MORU | 34 | 34 |
| *L. interrogans^a^* | Undesignated | C10010 | Thailand | 2004 | Bandicoot rat | NIH | 34 | 34 |
| *L. interrogans^a^* | Undesignated | C10064 | Thailand | 2004 | Bandicoot rat | NIH | 34 | 34 |
| *L. interrogans^a^* | Undesignated | C30015 | Thailand | 2004 | Bandicoot rat | NIH | 34 | 34 |
| *L. interrogans^a^* | Undesignated | C3003 | Thailand | 2004 | Bandicoot rat | NIH | 34 | 34 |
| *L. interrogans^a^* | Undesignated | C3004 | Thailand | 2004 | Bandicoot rat | NIH | 34 | 34 |
| *L. interrogans^a^* | Undesignated | C3005 | Thailand | 2004 | Bandicoot rat | NIH | 34 | 34 |
| *L. interrogans^a^* | Undesignated | H3 | Thailand | 2004 | Human | NIH | 34 | 34 |
| *L. interrogans^a^* | Undesignated | H4 | Thailand | 2004 | Human | NIH | 34 | 34 |
| *L. interrogans^a^* | Undesignated | P3010 | Thailand | 2004 | Bandicoot rat | NIH | 34 | 34 |
| *L. interrogans* | Autumnalis | UI08596 | Laos | 2006 | Human | WTMORC | 34 | 34 |
| *L. interrogans* | Javanica | UI08704 | Laos | 2006 | Human | WTMORC | 34 | 34 |
| *L. interrogans* | Autumnalis | UI12268 | Laos | 2008 | Human | WTMORC | 34 | 34 |
| *L. interrogans* | Autumnalis | UI12539 | Laos | 2008 | Human | WTMORC | 34 | 34 |
| *L. interrogans* | Autumnalis | UI12627 | Laos | 2008 | Human | WTMORC | 34 | 34 |
| *L. interrogans* | Autumnalis | UI12830 | Laos | 2008 | Human | WTMORC | 34 | 34 |
| *L. interrogans* | Autumnalis | UI13005 | Laos | 2008 | Human | WTMORC | 34 | 34 |
| *L. interrogans* | Autumnalis | UI13016 | Laos | 2008 | Human | WTMORC | 34 | 34 |
| *L. interrogans^a^* | Medanensis | Hond HC | Indonesia | 1929 | Dog | WCCRRL | 35 | 35 |
| *L. interrogans^a^* | Undesignated | Aki B (NIJ) | Japan |  |  | WCCRRL | 35 | 138 |
| *L. interrogans^a^* | Undesignated | FPW1039 | Thailand | 2004 | Human | MORU | 35 | 139 |
| *L. interrogans^a^* | Hebdomadis | Hebdomadis | Japan | 1916 | Human | NIH | 36 | 36 |
| *L. interrogans^a^* | Canicola | Hond Utrecht IV | Netherlands | 1931 | Dog | NIH | 37 | 37 |
| *L. interrogans^a^* | Portlandvere | MY 1039 | Jamaica | 1982^b^ | Human | WCCRRL | 37 | 37 |
| *L. interrogans* | Undesignated | J180 | China | 2009 | Canine | NICDCP | 37 | 37 |
| *L. interrogans^a^* | Canicola | UT479 | Thailand | 2005 | Human | MORU | 37 | 37 |
| *L. interrogans^a^* | Pyrogenes | UD009 | Thailand | 2003 | Human | MORU | 37 | 37 |
| *L. interrogans^a^* | Canicola | UI08414 | Laos | 2006 | Human | WTMORC | 37 | 37 |
| *L. interrogans* | Undesignated | UI09600 | Laos | 2007 | Human | WTMORC | 37 | 37 |
| *L. interrogans* | Undesignated | UI09661 | Laos | 2007 | Human | WTMORC | 37 | 37 |
| *L. interrogans^a^* | Pomona | Pomona | Australia | 1936 | Human | WCCRRL | 37 | 140 |
| *L. interrogans^a^* | Guaratuba | An 7705 | Brazil | 1975^b^ | Opossum | WCCRRL | 37 | 140 |
| *L. interrogans* | Undesignated | R235 | Sri Lanka | 2006 | Human | Mark Bailey | 37 | 140 |
| *L. interrogans^a^* | Gem | Simon | Sri Lanka | 1966 | Human | WCCRRL | 38 | 38 |
| *L. interrogans^a^* | Pomona | UT364 | Thailand | 2005 | Human | MORU | 38 | 38 |
| *L. interrogans^a^* | Undesignated | Aki C (NIJ) | Japan |  |  | WCCRRL | 39 | 39 |
| *L. interrogans^a^* | Undesignated | UT126 | Thailand | 2003 | Human | MORU | 40 | 40 |
| *L. interrogans^a^* | Autumnalis | L0594 | Thailand | 2001 | Human | MORU | 41 | 41 |
| *L. interrogans^a^* | Bataviae | L1111 | Thailand | 2002 | Human | MORU | 42 | 42 |
| *L. interrogans^a^* | Bataviae | L1085 | Thailand | 2002 | Human | MORU | 42 | 42 |
| *L. interrogans^a^* | Weerasinghe | Weerasinghe | Sri Lanka | 1965-1966 | Human | WCCRRL | 43 | 43 |
| *L. interrogans^a^* | Geyaweera | Geyaweera | Sri Lanka | 1965 | Human | WCCRRL | 44 | 44 |
| *L. interrogans^a^* | Undesignated | FPW1024 | Thailand | 2004 | Human | MORU | 45 | 45 |
| *L. interrogans^a^* | Bataviae | UT229 | Thailand | 2004 | Human | MORU | 46 | 46 |
| *L. interrogans^a^* | Bataviae | UT234 | Thailand | 2004 | Human | MORU | 46 | 46 |
| *L. interrogans^a^* | Undesignated | L0996 | Thailand | 2002 | Human | MORU | 46 | 46 |
| *L. interrogans^a^* | Undesignated | UT053 | Thailand | 2003 | Human | MORU | 46 | 46 |
| *L. interrogans^a^* | Medanensis | L0448 | Thailand | 2001 | Human | MORU | 46 | 46 |
| *L. interrogans^a^* | Medanensis | L0887 | Thailand | 2002 | Human | MORU | 46 | 46 |
| *L. interrogans^a^* | Medanensis | L0941 | Thailand | 2002 | Human | MORU | 46 | 46 |
| *L. interrogans^a^* | Undesignated | FPW2026 | Thailand | 2004 | Human | MORU | 47 | 47 |
| *L. interrogans^a^* | Camlo | LT 64-67 | Vietnam | 1967 | Human | WCCRRL | 48 | 48 |
| *L. interrogans^a^* | Pyrogenes | L0374 | Thailand | 2001 | Human | MORU | 49 | 49 |
| *L. interrogans^a^* | Pyrogenes | L0387 | Thailand | 2001 | Human | MORU | 49 | 49 |
| *L. interrogans^a^* | Pyrogenes | L0443 | Thailand | 2001 | Human | MORU | 49 | 49 |
| *L. interrogans^a^* | Pyrogenes | L0784 | Thailand | 2002 | Human | MORU | 49 | 49 |
| *L. interrogans* | Pyrogenes | R122 | Sri Lanka | 2006 | Human | Mark Bailey | 49 | 49 |
| *L. interrogans* | Pyrogenes | R150 | Sri Lanka | 2006 | Human | Mark Bailey | 49 | 49 |
| *L. interrogans* | Pyrogenes | R168 | Sri Lanka | 2006 | Human | Mark Bailey | 49 | 49 |
| *L. interrogans^a^* | Undesignated | C10069 | Thailand | 2004 | Black rat | NIH | 49 | 49 |
| *L. interrogans* | Pyrogenes | R166 | Sri Lanka | 2006 | Human | Mark Bailey | 49 | 49 |
| *L. interrogans* | Pyrogenes | R358 | Sri Lanka | 2006 | Human | Mark Bailey | 49 | 49 |
| *L. interrogans* | Pyrogenes | R480 | Sri Lanka | 2007 | Human | Mark Bailey | 49 | 49 |
| *L. interrogans^a^* | Bataviae | Swart | Indonesia | 1952 | Human | NIH | 50 | 50 |
| *L. interrogans^a^* | Losbanos | LT 101-69 | Philippines |  | Rat | WCCRRL | 50 | 50 |
| *L. interrogans^a^* | Australis | Ballico | Australia | 1934 | Human | WCCRRL | 51 | 51 |
| *L. interrogans^a^* | Kremastos | Kremastos | Australia | 1952 | Human | WCCRRL | 52 | 52 |
| *L. interrogans^a^* | Hawain | LT 62-68 | Papua New Guinea | 1971^b^ | Bandicoot | WCCRRL | 53 | 53 |
| *L. interrogans^a^* | Jonsis | Jones | Malaysia | 1957^b^ | Human | WCCRRL | 54 | 54 |
| *L. interrogans^a^* | Gurungi | Gurung | Malaysia | 1953-1955 | Human | WCCRRL | 55 | 55 |
| *L. interrogans^a^* | Ricardi | Richardson | Malaysia | 1953-1955 | Human | WCCRRL | 56 | 56 |
| *L. interrogans^a^* | Manilae | LT 398 | Philippines | 1957-1959 | Rat | WCCRRL | 57 | 57 |
| *L. interrogans^a^* | Roumanica | LM 294 | Romania | 1966 | House mouse | WCCRRL | 58 | 58 |
| *L. interrogans^a^* | Wolffi | 3705 | Indonesia | 1937 | Human | NIH | 58 | 58 |
| *L. interrogans^a^* | Bataviae | L1178 | Thailand | 2002 | Human | MORU | 59 | 59 |
| *L. interrogans^a^* | Bataviae | UT075 | Thailand | 2003 | Human | MORU | 59 | 59 |
| *L. interrogans^a^* | Carlos | C 3 | Philippines | 1970 | Toad | WCCRRL | 60 | 60 |
| *L. interrogans^a^* | Valbuzzi | Valbuzzi | Australia | 1955^b^ | Human | WCCRRL | 61 | 61 |
| *L. interrogans^a^* | Broomi | Patane | Australia | 1954 | Human | WCCRRL | 72 | 72 |
| *L. interrogans^a^* | Szwajizak | Szwajizak | Australia | 1952 | Human | WCCRRL | 73 | 73 |
| *L. interrogans* | Pyrogenes | R163 | Sri Lanka | 2006 | Human | Mark Bailey | 74 | 74 |
| *L. interrogans* | Pyrogenes | R205 | Sri Lanka | 2006 | Human | Mark Bailey | 75 | 75 |
| *L. interrogans* | Pyrogenes | R493 | Sri Lanka | 2007 | Human | Mark Bailey | 75 | 75 |
| *L. interrogans* | Pyrogenes | R601 | Sri Lanka | 2007 | Human | Mark Bailey | 75 | 75 |
| *L. interrogans* | Undesignated | R444 | Sri Lanka | 2007 | Human | Mark Bailey | 75 | 75 |
| *L. interrogans^a^* | Pyrogenes | 56605 | China |  | Human | NICDCP | 76 | 76 |
| *L. interrogans* | Undesignated | R437 | Sri Lanka | 2007 | Human | Mark Bailey | 76 | 76 |
| *L. interrogans* | Undesignated | R457 | Sri Lanka | 2007 | Human | Mark Bailey | 76 | 76 |
| *L. interrogans^a^* | Grippotyphosa | UI08368 | Laos | 2006 | Human | WTMORC | 77 | 77 |
| *L. interrogans* | Grippotyphosa | UI08381 | Laos | 2006 | Human | WTMORC | 78 | 78 |
| *L. interrogans^a^* | Bataviae | UI08561 | Laos | 2006 | Human | WTMORC | 79 | 79 |
| *L. interrogans^a^* | Undesignated | R499 | Sri Lanka | 2007 | Human | Mark Bailey | 80 | 80 |
| *L. interrogans^a^* | Autumnalis | UI08440 | Laos | 2006 | Human | WTMORC | 81 | 81 |
| *L. interrogans^a^* | Grippotyphosa | UI08434 | Laos | 2006 | Human | WTMORC | 82 | 82 |
| *L. interrogans^a^* | Autumnalis | UI12621 | Laos | 2008 | Human | WTMORC | 83 | 83 |
| *L. interrogans^a^* | Autumnalis | UI12758 | Laos | 2008 | Human | WTMORC | 84 | 84 |
| *L. interrogans^a^* | Grippotyphosa | UI12764 | Laos | 2008 | Human | WTMORC | 85 | 85 |
| *L. interrogans^a^* | Grippotyphosa | UI12769 | Laos | 2008 | Human | WTMORC | 86 | 86 |
| *L. interrogans^a^* | Autumnalis | UI13372 | Laos | 2008 | Human | WTMORC | 87 | 87 |
| *L. interrogans^a^* | Pyrogenes | EGY-AFI-MAL-047 | Egypt | 2005 | Human | NCEZID | 88 | 88 |
| *L. interrogans^a^* | Undesignated | 7751 | China | 1977 | Human | NICDCP | 89 | 89 |
| *L. interrogans^a^* | Hebdomadis | 9073 | China | 1990 | Human | NICDCP | 90 | 90 |
| *L. interrogans^a^* | Fortbragg | 9078 | China | 1990 | Human | NICDCP | 91 | 91 |
| *L. interrogans^a^* | Linhai | 9087 | China | 1990 | Human | NICDCP | 92 | 92 |
| *L. interrogans^a^* | Australis | 9116 | China | 1991 | Rattus nitidus | NICDCP | 93 | 93 |
| *L. interrogans^a^* | Nanla | 9188 | China | 1991 | Human | NICDCP | 94 | 91 |
| *L. interrogans^a^* | Autumnalis | 56606 | China |  | Human | NICDCP | 95 | 95 |
| *L. interrogans^a^* | Paidjan | 56612 | China |  | Human | NICDCP | 96 | 96 |
| *L. interrogans^a^* | Wolffi | 56635 | China |  | Human | NICDCP | 97 | 97 |
| *L. interrogans^a^* | Honghe | 81244 | China | 1981 | Human | NICDCP | 98 | 98 |
| *L. interrogans^a^* | Undesignated | 81522 | China | 1981 | Human | NICDCP | 99 | 99 |
| *L. interrogans^a^* | Undesignated | 200040 | China | 2000 | Human | NICDCP | 100 | 89 |
| *L. interrogans^a^* | Undesignated | 200305 | China | 2003 | Human | NICDCP | 101 | 101 |
| *L. interrogans^a^* | Undesignated | 200509 | China | 2005 | Apodemus chevrieri | NICDCP | 102 | 102 |
| *L. interrogans^a^* | Undesignated | A05D31 | China | 2005 | Apodemus agrarius | NICDCP | 103 | 36 |
| *L. interrogans^a^* | Undesignated | A05D39 | China | 2005 | Apodemus agrarius | NICDCP | 104 | 18 |
| *L. interrogans^a^* | Undesignated | J39 | China | 2006 | Human | NICDCP | 105 | 105 |
| *L. interrogans* | Undesignated | J100 | China | 2007 | Apodemus agrarius | NICDCP | 106 | 106 |
| *L. interrogans^a^* | Undesignated | J42 | China | 2006 | Human | NICDCP | 106 | 106 |
| *L. interrogans^a^* | Undesignated | J7 | China | 2005 | Rattus tanezumi | NICDCP | 107 | 107 |
| *L. interrogans^a^* | Undesignated | J9 | China | 2006 | Rattus flavipectus | NICDCP | 108 | 108 |
| *L. interrogans^a^* | Undesignated | Shuang4 | China | 2005 | Frog | NICDCP | 109 | 109 |
| *L. interrogans^a^* | Grippotyphosa | EGY-AFI-MAL-058 | Egypt |  | Human | NCEZID | 111 | 111 |
| *L. interrogans^a^* | Bulgarica | Malika | India |  | Human | NCEZID | 112 | 112 |
| *L. interrogans^a^* | Undesignated | UD-13 | Thailand |  | Human | NCEZID | 113 | 113 |
| *L. interrogans^a^* | Undesignated | D-MZ07-2E | Japan | 2007 | Dog | Nobuo Koizumi | 118 | 118 |
| *L. interrogans^a^* | Undesignated | D-MZ07-6E | Japan | 2007 | Dog | Nobuo Koizumi | 119 | 119 |
| *L. interrogans^a^* | Undesignated | D-FO11-11K | Japan | 2011 | Dog | Nobuo Koizumi | 120 | 120 |
| *L. interrogans* | Undesignated | H0728 | China | 2007 | Apodemus agrarius | NICDCP | 128 | 128 |
| *L. interrogans* | Undesignated | H0754 | China | 2007 | Apodemus agrarius | NICDCP | 128 | 128 |
| *L. interrogans* | Undesignated | H0775 | China | 2007 | House mouse | NICDCP | 129 | 129 |
| *L. interrogans* | Undesignated | 9086 | China | 1990 | Human | NICDCP | 130 | 130 |
| *L. interrogans* | Undesignated | J109 | China | 2007 | Rattus norvegicus | NICDCP | 131 | 131 |
| *L. interrogans* | Undesignated | As-AK09-25 | Japan | 2009 | Apodemus speciosus | Nobuo Koizumi | 132 | 132 |
| *L. interrogans* | Undesignated | NIID9 | Malaysia | 2007 | Human | Nobuo Koizumi | 133 | 133 |
| *L. interrogans* | Undesignated | NIID10 | Malaysia | 2010 | Human | Nobuo Koizumi | 134 | 134 |
| *L. interrogans* | Undesignated | E93 | Philippines | 2007 | Rat | Nobuo Koizumi | 135 | 135 |
| *L. interrogans* | Undesignated | K1-5 | Philippines | 2006 | Rat | Nobuo Koizumi | 135 | 135 |
| *L. kirschneri^a^* | Grippotyphosa | Moskva V | Russia | 1928^b^ | Human | WCCRRL | 62 | 62 |
| *L. kirschneri^a^* | Bim | 1051 | Barbados | 1985^b^ | Dog | WCCRRL | 63 | 63 |
| *L. kirschneri* | Bafani | Bafani | Zaire | 1946 | Human | KIT | 63 | 141 |
| *L. kirschneri^a^* | Erinaceiauriti | Erinaceus Auritus 670 | Russia | 1951^b^ | Hedgehog | WCCRRL | 64 | 64 |
| *L. kirschneri^a^* | Mwogolo | Mwogolo | Zaire | 1938 | Human | WCCRRL | 65 | 65 |
| *L. kirschneri^a^* | Lambwe | Lambwe | Kenya | 1968 | Unstriped grass rat | WCCRRL | 66 | 66 |
| *L. kirschneri^a^* | Bulgarica | Nicolaevo | Bulgaria | 1951 | Human | WCCRRL | 67 | 67 |
| *L. kirschneri^a^* | Grippotyphosa | UT130 | Thailand | 2003 | Human | MORU | 68 | 68 |
| *L. kirschneri^a^* | Undesignated | H1 | Thailand | 2004 | Human | NIH | 68 | 68 |
| *L. kirschneri^a^* | Butembo | Butembo | Zaire | 1946^b^ | Human | KIT | 69 | 69 |
| *L. kirschneri^a^* | Cynopteri | 3522 C | Indonesia | 1938 | Bat | NIH | 70 | 70 |
| *L. kirschneri* | Kunming | K 5 | China | 1984^b^ | Mouse | KIT | 70 | 70 |
| *L. kirschneri^a^* | Undesignated | H2 | Thailand | 2004 | Human | NIH | 71 | 71 |
| *L. kirschneri^a^* | Grippotyphosa | M631 | Croatia | 2005 | Microtus arvalis | NCEZID | 110 | 110 |
| *L. kirschneri* | Valbuzzi | Duyster |  |  |  | KIT | 110 | 110 |
| *L. kirschneri* | Vanderhoedeni | Kipod 179 | Israel | 1969^b^ | Long-eared hedgehog | KIT | 110 | 110 |
| *L. kirschneri^a^* | Tsaratsovo | B 81/7 | Bulgaria | 1962 | Harvest mouse | NCEZID | 115 | 115 |
| *L. kirschneri^a^* | Ratnapura | Wumalasena | Sri Lanka | 1965-1966 | Human | KIT | 116 | 116 |
| *L. kirschneri^a^* | Mozdok | 5621 | Russia | 1961 | Field vole | KIT | 117 | 117 |
| *L. kirschneri* | Ramisi | Musa | Kenya | 1971 | Human | KIT | 121 | 121 |
| *L. kirschneri* | Bogvere | LT 60-69 | Jamaica |  | Rat | KIT | 122 | 122 |
| *L. kirschneri* | Ndambari | Ndambari | Zaire | 1938 | Human | KIT | 122 | 122 |
| *L. kirschneri* | Kambale | Kambale | Zaire | 1955-1956 | Human | KIT | 123 | 123 |
| *L. kirschneri* | Galtoni | LT 1014 | Argentina | 1964 | Cow | KIT | 124 | 124 |
| *L. kirschneri* | Kabura | Kabura | Zaire | 1938-1946 | Human | KIT | 125 | 125 |
| *L. kirschneri* | Kamituga | Kamituga | Zaire | 1939 | Human | KIT | 126 | 126 |
| *L. kirschneri* | Ndahambukuje | Ndahambukuje | Zaire | 1938 | Human | KIT | 127 | 127 |
| *L. kirschneri* | Undesignated | SU-HO11-5F5 | Japan | 2011 | Shrew | Kyle Taylor | 136 | 136 |
| *L. kirschneri* | Undesignated | SU-HO11-7F1 | Japan | 2011 | Shrew | Kyle Taylor | 136 | 136 |
| *L. borgpetersenii* | Mini | Sari | Italy | 1940 | Human | KIT | Untypeable | 142 |
| *L. borgpetersenii* | Javanica | L0066 | Thailand | 2003 | Human | MORU | Untypeable | 143 |
| *L. borgpetersenii* | Javanica | L0864 | Thailand | 2002 | Human | MORU | Untypeable | 143 |
| *L. borgpetersenii* | Javanica | MK146 | Thailand | 2004 | Human | MORU | Untypeable | 143 |
| *L. borgpetersenii* | Javanica | Veldrat Batavia 46 | Indonesia | 1938 | Field rat | KIT | Untypeable | 143 |
| *L. borgpetersenii* | Undesignated | RR-KS-KC08-2B | Japan | 2008 | Rat | Nobuo Koizumi | Untypeable | 143 |
| *L. borgpetersenii* | Undesignated | RR-OW-MY10-12 | Japan | 2010 | Rat | Nobuo Koizumi | Untypeable | 143 |
| *L. borgpetersenii* | Undesignated | RR-OW05-45 | Japan | 2005 | Rat | Nobuo Koizumi | Untypeable | 143 |
| *L. borgpetersenii* | Zhenkang | L 82 | China | 1988^b^ | House rat | KIT | Untypeable | 143 |
| *L. borgpetersenii* | Javanica | UI09931 | Laos | 2007 | Human | WTMORC | Untypeable | 143 |
| *L. borgpetersenii* | Undesignated | R010 | Sri Lanka | 2006 | Human | Mark Bailey | Untypeable | 144 |
| *L. borgpetersenii* | Ceylonica | Piyasena | Sri Lanka | 1964 | Human | KIT | Untypeable | 144 |
| *L. borgpetersenii* | Undesignated | L0101 | Thailand | 2000 | Human | MORU | Untypeable | 144 |
| *L. borgpetersenii* | Javanica | UI09149 | Laos | 2007 | Human | WTMORC | Untypeable | 144 |
| *L. borgpetersenii* | Poi | Poi | Italy | 1941 | Human | KIT | Untypeable | 145 |
| *L. borgpetersenii* | Sorexjalna | Sorex Jalna | Czechoslovakia | 1953 | Shrew | KIT | Untypeable | 146 |
| *L. borgpetersenii* | Undesignated | SU-HO11-5F3 | Japan | 2011 | Shrew | Nobuo Koizumi | Untypeable | 146 |
| *L. borgpetersenii* | Undesignated | SU-HO11-9F3 | Japan | 2011 | Shrew | Nobuo Koizumi | Untypeable | 146 |
| *L. borgpetersenii* | Jules | Jules | Zaire | 1952-1953 | Human | KIT | Untypeable | 147 |
| *L. borgpetersenii* | Kisuba | Kisuba | Zaire | 1956^b^ | Human | KIT | Untypeable | 148 |
| *L. borgpetersenii* | Ballum | Mus 127 | Denmark | 1944^b^ | Field mouse | KIT | Untypeable | 149 |
| *L. borgpetersenii* | Castellonis | Castellon 3 | Spain | 1953-1955 | Wood mouse | KIT | Untypeable | 149 |
| *L. borgpetersenii* | Undesignated | MC-OW08-2 | Japan | 2008 | Mouse | Nobuo Koizumi | Untypeable | 149 |
| *L. borgpetersenii* | Balcanica | 1627 Burgas | Bulgaria | 1958 | Human | KIT | Untypeable | 150 |
| *L. borgpetersenii* | Kwale | Julu | Kenya | 1968 | Human | KIT | Untypeable | 151 |
| *L. borgpetersenii* | Hardjo-bovis | JB197 | USA |  | Beef steer | GenBank | Untypeable | 152 |
| *L. borgpetersenii* | Hardjo-bovis | L550 | Australia |  | Human | GenBank | Untypeable | 152 |
| *L. borgpetersenii* | Hardjo-bovis | Sponselee | Netherlands |  | Bovine | KIT | Untypeable | 152 |
| *L. borgpetersenii* | Tarassovi | Perepelitsin | Russia | 1941^b^ | Human | KIT | Untypeable | 153 |
| *L. borgpetersenii* | Tunis | P 2/65 | Tunisia | 1965 | Pig | KIT | Untypeable | 153 |
| *L. borgpetersenii* | Kenya | Nijenga | Kenya | 1968 | Pouched Rat | KIT | Untypeable | 154 |
| *L. borgpetersenii* | Polonica | 493 Poland | Poland | 1957 | Hedgehog | KIT | Untypeable | 155 |
| *L. borgpetersenii* | Nyanza | Kibos | Kenya | 1969 | Human | KIT | Untypeable | 156 |
| *L. borgpetersenii* | Undesignated | R116 | Sri Lanka | 2006 | Human | Mark Bailey | Untypeable | 157 |
| *L. borgpetersenii* | Undesignated | MRB-HO11-17 | Japan | 2011 | Mouse | Nobuo Koizumi | Untypeable | 197 |
| *L. borgpetersenii* | Undesignated | MRM-HO09-40 | Japan | 2009 | Mouse | Nobuo Koizumi | Untypeable | 197 |
| *L. alexanderi* | Manhao 3 | L60 | China | 1984^b^ |  | NCEZID | Untypeable | 158 |
| *L. alexanderi* | Mengla | A 85 | China | 1970 | Human | KIT | Untypeable | 159 |
| *L. alexanderi* | Yunnan | A 10 | China | 1962 | Human | NCEZID | Untypeable | 160 |
| *L. alexanderi* | Nanding | M 6901 | China |  | Human | NCEZID | Untypeable | 161 |
| *L. alexanderi* | Manzhuang | A 23 | China | 1962 | Human | KIT | Untypeable | 162 |
| *L. noguchii* | Nicaragua | 1011 | Nicaragua | 1967 | Weasel | NCEZID | Untypeable | 163 |
| *L. noguchii* | Argentiniensis | Peludo | Argentina | 1963 | Armadillo | KIT | Untypeable | 164 |
| *L. noguchii* | Proechimys | 1161 U | Panama | 1982^b^ | Spiny rat | KIT | Untypeable | 165 |
| *L. noguchii* | Carimagua | 9160 | Argentina |  |  | KIT | Untypeable | 166 |
| *L. noguchii* | Fortbragg | Fort Bragg | USA | 1944 | Human | WCCRRL | Untypeable | 167 |
| *L. noguchii* | Undesignated | 2007001578 | USA |  |  | NCEZID | Untypeable | 168 |
| *L. noguchii* | Louisiana | LSU 1945 | USA | 1964^b^ | Armadillo | KIT | Untypeable | 169 |
| *L. noguchii* | Huallaga | M 7 | Peru | 1970 | Opossum | KIT | Untypeable | 170 |
| *L. noguchii* | Panama | CZ 214 K | Panama | 1962 | Opossum | NCEZID | Untypeable | 171 |
| *L. santarosai* | Beye | 1537 U | Panama | 1960-1962 | Spiny rat | KIT | Untypeable | 172 |
| *L. santarosai* | Babudieri | CI 40 | Peru |  | Pig | KIT | Untypeable | 173 |
| *L. santarosai* | Balboa | 735 U | Panama | 1966 | Spiny rat | KIT | Untypeable | 174 |
| *L. santarosai* | Kobbe | CZ 320 | Panama | 1962 | Spiny rat | KIT | Untypeable | 175 |
| *L. santarosai* | Canalzonae | CZ 188 | Panama | 1964 | Spiny rat | KIT | Untypeable | 176 |
| *L. santarosai* | Fluminense | Aa 3 | Brazil | 1970 | Field mouse | KIT | Untypeable | 177 |
| *L. santarosai* | Alice | Alice | Sri Lanka |  | Human | WCCRRL | Untypeable | 178 |
| *L. santarosai* | Guaricura | Bov. G | Brazil | 1962-1968 | Cow | KIT | Untypeable | 179 |
| *L. santarosai* | Borincana | HS 622 | Puerto Rico | 1951 | Human | KIT | Untypeable | 180 |
| *L. santarosai* | Atchafalaya | LSU 1013 | USA | 1963^b^ | Opossum | KIT | Untypeable | 181 |
| *L. weilii* | Mengma | S 590 | China | 1986 | Human | NCEZID | Untypeable | 182 |
| *L. weilii* | Menrun | A 102 | China | 1970 | Human | NCEZID | Untypeable | 182 |
| *L. weilii* | Undesignated | LNT1234 | Laos | 2009 | Human | WTMORC | Untypeable | 183 |
| *L. weilii* | Mengdeng | M 6906 | China | 1969 | Human | NCEZID | Untypeable | 184 |
| *L. weilii* | Celledoni | Celledoni | Australia | 1952 | Human | KIT | Untypeable | 185 |
| *L. weilii* | Coxi | Cox | Malaysia | 1953-1956 | Human | WCCRRL | Untypeable | 186 |
| *L. weilii* | Mengdeng | LNT1194 | Laos | 2009 | Human | WTMORC | Untypeable | 187 |
| *L. weilii* | Vughia | LT 89-68 | Vietnam | 1971^b^ | Human | NCEZID | Untypeable | 188 |
| *L. weilii* | Mengdeng | UI14631 | Laos | 2009 | Human | WTMORC | Untypeable | 189 |
| *L. weilii* | Mengdeng | UI13098 | Laos | 2008 | Human | WTMORC | Untypeable | 190 |
| *L. weilii* | Sarmin | Sarmin | Indonesia | 1930 | Human | NIH | Untypeable | 191 |
| *L. weilii* | Topaz | 94-79970/3 | Australia |  | Bovine | WCCRRL | Untypeable | 192 |
| *L. weilii* | Hekou | H 27 | China | 1964 | Human | WCCRRL | Untypeable | 193 |
| *L. weilii* | Menglian | S 621 | China | 1981 | Human | WCCRRL | Untypeable | 194 |
| *L. weilii* | Unipertama | K2-1 | Indonesia |  | Bovine | WCCRRL | Untypeable | 195 |
| *L. weilii* | Langati | M 39090 | Malaysia |  | Human | WCCRRL | Untypeable | 196 |

^a^ One hundred and ninety-nine isolates reported previously by Thaipadungpanit et al*.* and/or submitted previously to MLST database ([www.MLST.net](http://www.MLST.net/)).

^b^ Year of valid description rather than isolation.

^c^ Source of DNA and cultures: MORU, Mahidol-Oxford Tropical Medicine Research Unit, Mahidol University, Thailand; WCCRRL, WHO/FOA/OIE/ Collaborating Center for Reference and Research on Leptospirosis, Queensland Health Forensic and Scientific Services, Australia; NIH, Bureau of Emerging Infectious Disease, Ministry of Public Health, Thailand; KIT, Biomedical Research, WHO/FAO/OIE and National Leptospirosis Reference Centre, Amsterdam, Netherlands; NCEZID, National Center for Emerging and Zoonotic Infectious Diseases, Centers for Diseases Control and Prevention, USA; WTMORC, Wellcome Trust-Mahosot Hospital-Oxford University Tropical Medicine Research Collaboration, Laos; NICDCP, Department of Leptospirosis, National Institute for Communicable Disease Control and Prevention, Chinese Center for Disease Control and Prevention, China.

^d^ Typed using MLST described by Thaipadungpanit et al.

^e^ Typed using modified MLST scheme described in this study.
