# Supplementary material for: A Single Multilocus Sequence Typing (MLST) Scheme for Seven Pathogenic Leptospira Species
Source: PLoS Negl Trop Dis. 2013 Jan 24;7(1):e1954. doi: 10.1371/journal.pntd.0001954 (PMC3554523; doi:10.1371/journal.pntd.0001954)
Supplement: Table S2 — Accession numbers for rrs fragments sequenced in this study. (DOC) [file pntd.0001954.s004.doc]

**Table S2. Accession numbers for *rrs* fragments sequenced in this study.**

| **Species** | **Serovar** | **Strain** | **GenBank ID** |
| --- | --- | --- | --- |
| *L. alexanderi* | Manzhuang | A 23 | AY996803.1 |
| *L. alexanderi* | Mengla | A 85 | DQ991481.1 |
| *L. alexanderi* | Manhao | L60 | AY631880.1 |
| *L. alexanderi* | Nanding | M 6901 | AY996804.1 |
| *L. borgpetersenii* | Javanica | L0066 | EF537005 |
| *L. borgpetersenii* | Javanica | L0864 | EF537006 |
| *L. borgpetersenii* | Undesignated | MC-OW08-2 | JQ906669 |
| *L. borgpetersenii* | Undesignated | MRB-HO11-17 | JX089386 |
| *L. borgpetersenii* | Undesignated | MRM-HO09-40 | JX089387 |
| *L. borgpetersenii* | Undesignated | R116 | JX040543 |
| *L. borgpetersenii* | Undesignated | RR-KS-KC08-2B | JQ906662 |
| *L. borgpetersenii* | Undesignated | RR-OW-MY10-12 | JQ906664 |
| *L. borgpetersenii* | Undesignated | RR-OW05-45 | JQ906663 |
| *L. borgpetersenii* | Mini | Sari | AM050573.1 |
| *L. borgpetersenii* | Undesignated | SU-HO11-5F3 | JQ906667 |
| *L. borgpetersenii* | Undesignated | SU-HO11-9F3 | JQ906668 |
| *L. borgpetersenii* | Undesignated | UI09149 | JQ906666 |
| *L. borgpetersenii* | Undesignated | UI09931 | JQ906665 |
| *L. interrogans* | Undesignated | 7751 | JQ906631 |
| *L. interrogans* | Hebdomadis | 9073 | JQ906633 |
| *L. interrogans* | Fortbragg | 9078 | JQ906634 |
| *L. interrogans* | Undesignated | 9086 | JQ906651 |
| *L. interrogans* | Linhai | 9087 | JQ906636 |
| *L. interrogans* | Australis | 9116 | JQ906637 |
| *L. interrogans* | Nanla | 9188 | JQ906635 |
| *L. interrogans* | Undesignated | 81522 | JQ906638 |
| *L. interrogans* | Undesignated | 200040 | JQ906632 |
| *L. interrogans* | Undesignated | 200305 | JQ906639 |
| *L. interrogans* | Undesignated | 200509 | JQ906640 |
| *L. interrogans* | Undesignated | A05D31 | JQ906617 |
| *L. interrogans* | Undesignated | A05D39 | JQ906616 |
| *L. interrogans* | Undesignated | As-AK09-25 | JQ906653 |
| *L. interrogans* | Undesignated | D-FO11-11K | JQ906647 |
| *L. interrogans* | Undesignated | D-MZ07-2E | JQ906645 |
| *L. interrogans* | Undesignated | D-MZ07-6E | JQ906646 |
| *L. interrogans* | Undesignated | E93 | JQ906656 |
| *L. interrogans* | Undesignated | FPW1024 | EF536986 |
| *L. interrogans* | Undesignated | FPW2026 | EF536989 |
| *L. interrogans* | Undesignated | H0728 | JQ906648 |
| *L. interrogans* | Undesignated | H0754 | JQ906649 |
| *L. interrogans* | Undesignated | H0775 | JQ906650 |
| *L. interrogans* | Undesignated | J109 | JQ906652 |
| *L. interrogans* | Undesignated | J39 | JQ906641 |
| *L. interrogans* | Undesignated | J42 | JQ906642 |
| *L. interrogans* | Undesignated | J7 | JQ906643 |
| *L. interrogans* | Undesignated | K1-5 | JQ906657 |
| *L. interrogans* | Kremastos | Kremastos | FJ154564.1 |
| *L. interrogans* | Autumnalis | L0013 | EF536978 |
| *L. interrogans* | Pyrogenes | L0374 | EF536990 |
| *L. interrogans* | Pyrogenes | L0387 | EF536991 |
| *L. interrogans* | Autumnalis | L0594 | EF536983 |
| *L. interrogans* | Grippotyphosa | L1006 | EF536975 |
| *L. interrogans* | Undesignated | L1085 | EF536984 |
| *L. interrogans* | Bataviae | L1111 | EF536985 |
| *L. interrogans* | Bataviae | L1178 | EF536994 |
| *L. interrogans* | Autumnalis | LP101 | EF536976 |
| *L. interrogans* | Undesignated | NIID10 | JQ906655 |
| *L. interrogans* | Undesignated | NIID9 | JQ906654 |
| *L. interrogans* | Undesignated | R163 | JQ906618 |
| *L. interrogans* | Pyrogenes | R205 | JQ906619 |
| *L. interrogans* | Undesignated | R437 | JQ906620 |
| *L. interrogans* | Undesignated | R499 | JQ906623 |
| *L. interrogans* | Autumnalis | RY021 | EF536979 |
| *L. interrogans* | Undesignated | Shuang4 | JQ906644 |
| *L. interrogans* | Pyrogenes | UD009 | EF536980 |
| *L. interrogans* | Grippotyphosa | UI08368 | JQ906621 |
| *L. interrogans* | Grippotyphosa | UI08381 | JQ906696 |
| *L. interrogans* | Grippotyphosa | UI08434 | JQ906625 |
| *L. interrogans* | Undesignated | UI08440 | JQ906624 |
| *L. interrogans* | Bataviae | UI08561 | JQ906622 |
| *L. interrogans* | Undesignated | UI12621 | JQ906626 |
| *L. interrogans* | Undesignated | UI12758 | JQ906627 |
| *L. interrogans* | Grippotyphosa | UI12764 | JQ906628 |
| *L. interrogans* | Grippotyphosa | UI12769 | JQ906629 |
| *L. interrogans* | Undesignated | UI13372 | JQ906630 |
| *L. interrogans* | Undesignated | UT053 | EF536988 |
| *L. interrogans* | Bataviae | UT075 | EF536993 |
| *L. interrogans* | Undesignated | UT126 | EF536982 |
| *L. interrogans* | Bataviae | UT229 | EF536987 |
| *L. interrogans* | Pomona | UT364 | EF536981 |
| *L. kirschneri* | Undesignated | H1 | EF536996 |
| *L. kirschneri* | Undesignated | H2 | EF536998 |
| *L. kirschneri* | Grippotyphosa | Moskva V | EF536995 |
| *L. kirschneri* | Ramisi | Musa | FJ154573.1 |
| *L. kirschneri* | Undesignated | SU-HO11-5F5 | JQ906658 |
| *L. kirschneri* | Undesignated | SU-HO11-7F1 | JQ906659 |
| *L. kirschneri* | Grippotyphosa | UT130 | EF536997 |
| *L. noguchii* | Cascata | Cascata^*^ | EU349495.1 |
| *L. noguchii* | Panama | CZ 214 K | FJ154582.1 |
| *L. noguchii* | Orleans | LSU 2580^*^ | FJ154588.1 |
| *L. santarosai* | Shermani | 1342 K^*^ | FJ154576.1 |
| *L. santarosai* | Shermani | 1343 K^*^ | Z21649.1 |
| *L. santarosai* | Georgia | LT 117^*^ | AY996805.1 |
| *L. santarosai* | Shermani | LT 821^*^ | AY631883.1 |
| *L. weilii* | Topaz | 94-79970/3 | DQ483058.1 |
| *L. weilii* | Undesignated | LNT1194 | JQ906671 |
| *L. weilii* | Undesignated | LNT1234 | JQ906670 |
| *L. weilii* | Vughia | LT 89-68 | FJ154590.1 |
| *L. weilii* | Undesignated | UI13098 | JQ906673 |
| *L. weilii* | Undesignated | UI14631 | JQ906672 |

^*^Not typed by MLST
