# Supplementary material for: A Single Multilocus Sequence Typing (MLST) Scheme for Seven Pathogenic Leptospira Species
Source: PLoS Negl Trop Dis. 2013 Jan 24;7(1):e1954. doi: 10.1371/journal.pntd.0001954 (PMC3554523; doi:10.1371/journal.pntd.0001954)
Supplement: Table S3 — dN/dS ratio of each MLST locus for 7 Leptospira species. (DOC) [file pntd.0001954.s005.doc]

**Table S3. dN/dS ratio of each MLST locus for 7 *Leptospira* species.**

|  | *glmU* | *pntA* | *sucA* | *tpiA* | *pfkB* | *mreA* | *caiB* |
| --- | --- | --- | --- | --- | --- | --- | --- |
| *L. alexanderi* | 0.0000 | 0.0000 | nd | 0.0000 | nd | 0.0000 | nd |
| *L. borgpetersenii* | 0.0459 | 0.0360 | 0.0000 | 0.0000 | 0.3158 | 0.1739 | 0.2465 |
| *L. interrogans* | 0.1438 | 0.0123 | 0.0373 | 0.1893 | 0.1139 | 0.0133 | 0.0409 |
| *L. kirschneri* | 0.1203 | 0.0313 | 0.4651 | 0.1496 | 0.1279 | 0.0377 | 0.2054 |
| *L. noguchii* | 0.1661 | 0.0158 | 0.0000 | 0.1074 | 0.1012 | 0.0000 | 0.0539 |
| *L. santarosai* | 0.1448 | 0.0511 | 0.0000 | 0.1789 | 0.2273 | 0.1843 | 0.3898 |
| *L. weilii* | 0.0723 | 0.0542 | 0.0000 | 0.1101 | 0.0186 | 0.0000 | 0.1401 |

nd, dN/dS ratio not determined as only a single unique allele identified
