# Supplementary material for: A Single Multilocus Sequence Typing (MLST) Scheme for Seven Pathogenic Leptospira Species
Source: PLoS Negl Trop Dis. 2013 Jan 24;7(1):e1954. doi: 10.1371/journal.pntd.0001954 (PMC3554523; doi:10.1371/journal.pntd.0001954)
Supplement: Table S4 — Serovars within individual sequence types (STs). (DOC) [file pntd.0001954.s006.doc]

**Table S4. Serovars within individual sequence types (STs).**

| **ST** | **No. of isolates** | **No. of serovars** | **Serovars (No. of isolates)** |
| --- | --- | --- | --- |
| 1 | 3 | 1 | Lai (2) |
| 18 | 2 | 1 | Grippotyphosa (1) |
| 27 | 2 | 1 | Autumnalis (1) |
| 34 | 61 | 1 | Autumnalis (52) |
| 36 | 2 | 1 | Hebdomadis (1) |
| 42 | 2 | 1 | Bataviae (2) |
| 49 | 11 | 1 | Pyrogenes (10) |
| 59 | 2 | 1 | Bataviae (2) |
| 68 | 2 | 1 | Grippotyphosa (1) |
| 75 | 4 | 1 | Pyrogenes (3) |
| 76 | 3 | 1 | Pyrogenes (1) |
| 146 | 3 | 1 | Sorexjalna (1) |
| 152 | 3 | 1 | Hardjo-bovis (3) |
| 17 | 4 | 2 | Copenhageni (2) and Icterohaemorrhagiae (2) |
| 20 | 2 | 2 | Haemolytica (1) and Hardjo (1) |
| 38 | 2 | 2 | Gem (1) and Pomona (1) |
| 46 | 7 | 2 | Batavie (2) and Medanensis (3) |
| 50 | 2 | 2 | Bataviae (1) and Losbanos (1) |
| 58 | 2 | 2 | Roumanica (1) and Wolffi (1) |
| 70 | 2 | 2 | Cynopteri (1) and Kunming (1) |
| 91 | 2 | 2 | Fortbragg (1) and Nanla (1) |
| 122 | 2 | 2 | Bogvere (1) and Ndambari (1) |
| 140 | 3 | 2 | Pomona (1) and Guaratuba (1) |
| 143 | 9 | 2 | Javanica (5) and Zhenkang (1) |
| 144 | 4 | 2 | Ceylonica (1) and Javanica (1) |
| 149 | 3 | 2 | Ballum (1) and Castellonis (1) |
| 153 | 2 | 2 | Tarassovi (1) and Tunis (1) |
| 182 | 2 | 2 | Mengma (1) and Menrun (1) |
| 24 | 3 | 3 | Bratislava (1), Jalna (1) and Muenchen (1) |
| 37 | 8 | 3 | Canicola (3), Portlandvere (1) and Pyrogenes (1) |
| 110 | 3 | 3 | Grippotyphosa (1), Valbuzzi (1) and Vanderhoedeni (1) |
