# Supplementary material for: A Single Multilocus Sequence Typing (MLST) Scheme for Seven Pathogenic Leptospira Species
Source: PLoS Negl Trop Dis. 2013 Jan 24;7(1):e1954. doi: 10.1371/journal.pntd.0001954 (PMC3554523; doi:10.1371/journal.pntd.0001954)
Supplement: Table S5 — Sequence types (STs) within individual serovars. (DOC) [file pntd.0001954.s007.doc]

**Table S5. Sequence types (STs) within individual serovars.**

| **Serovar** | **No. of isolates** | **No. of STs** | **STs within single serovars** |
| --- | --- | --- | --- |
| Canicola | 3 | 1 | 37 |
| Copenhageni | 2 | 1 | 17 |
| Hardjo-bovis | 3 | 1 | 152 |
| Icterohaemorrhagiae | 2 | 1 | 17 |
| Lai | 2 | 1 | 2 |
| Australis | 2 | 2 | 51, 93 |
| Bulgarica | 2 | 2 | 67, 112 |
| Fortbragg | 2 | 2 | 91, 167 |
| Hebdomadis | 2 | 2 | 36, 90 |
| Javanica | 6 | 2 | 143, 144 |
| Medanensis | 4 | 2 | 35, 46 |
| Nanla | 2 | 2 | 9, 91 |
| Paidjan | 2 | 2 | 2, 96 |
| Pomona | 2 | 2 | 38, 140 |
| Valbuzzi | 2 | 2 | 61, 110 |
| Wolffi | 2 | 2 | 58, 97 |
| Mengdeng | 4 | 4 | 184, 187, 189, 190 |
| Bataviae | 8 | 5 | 42, 46, 50,59, 79 |
| Pyrogenes | 18 | 7 | 13, 37, 49, 74, 75, 76, 88 |
| Autumnalis | 60 | 9 | 22, 27, 34, 41, 81, 83, 84, 87, 95 |
| Grippotyphosa | 10 | 10 | 18, 62, 68, 77, 78, 82, 85, 86, 110, 111 |
